# The Colonisation of Exotic Species Does Not Have to Trigger Faunal Homogenisation: Lessons from the Assembly Patterns of Arthropods on Oceanic Islands

**DOI:** 10.1371/journal.pone.0128276

**Published:** 2015-05-29

**Authors:** Margarita Florencio, Jorge M. Lobo, Pedro Cardoso, Mário Almeida-Neto, Paulo A. V. Borges

**Affiliations:** 1 CE3C —Centre for Ecology, Evolution and Environmental Changes, Azorean Biodiversity Group, Universidade dos Açores, Departamento de Ciências Agrárias, Angra do Heroísmo, Açores, Portugal; 2 CITA-A, Universidade dos Açores, Departamento de Ciências Agrárias, Angra do Heroísmo, Açores, Portugal; 3 Departamento de Ecologia, Instituto de Ciências Biológicas, Universidade Federal de Goiás, Goiânia, Goiás, Brazil; 4 Department of Biogeography and Global Change, Museo Nacional de Ciencias Naturales (CSIC), Madrid, Spain; 5 Finnish Museum of Natural History, University of Helsinki, Helsinki, Finland; University of Sydney, AUSTRALIA

## Abstract

Human-caused disturbances can lead to the extinction of indigenous (endemic and native) species, while facilitating and increasing the colonisation of exotic species; this increase can, in turn, promote the similarity of species compositions between sites if human-disturbed sites are consistently invaded by a regionally species-poor pool of exotic species. In this study, we analysed the extent to which epigean arthropod assemblages of four islands of the Azorean archipelago are characterised by nestedness according to a habitat-altered gradient. The degree of nestedness represents the extent to which less ubiquitous species occur in subsets of sites occupied by the more widespread species, resulting in an ordered loss/gain of species across environmental or ecological gradients. A predictable loss of species across communities while maintaining others may lead to more similar communities (i.e. lower beta-diversity). In contrast, anti-nestedness occurs when different species tend to occupy distinct sites, thus characterising a replacement of species across such gradients. Our results showed that an increase in exotic species does not promote assemblage homogenisation at the habitat level. On the contrary, exotic species were revealed as habitat specialists that constitute new and well-differentiated assemblages, even increasing the species compositional heterogeneity within human-altered landscapes. Therefore, contrary to expectations, our results show that both indigenous and exotic species established idiosyncratic assemblages within habitats and islands. We suggest that both the historical extinction of indigenous species in disturbed habitats and the habitat-specialised character of some exotic invasions have contributed to the construction of current assemblages.

## Introduction

Human land-use is a major driver of current changes in species assemblages across the world [1–2], promoting population declines and local extinctions of species and facilitating their replacement by invading exotic species [1]. In biological invasions, exotic species override geographical and ecological barriers becoming incorporated into indigenous (endemic and native) assemblages, and in turn transforming them [3]. These invasion processes are particularly accentuated on isolated oceanic islands, where exotic species usually occupy a wide range of niche opportunities with little competitive pressure in comparison to their native habitats [4–5]. Under these circumstances, exotic species may colonise human-altered landscapes where indigenous species might suffer a competitive disadvantage due to their putative intolerance to anthropogenic disturbances [6].

The widespread replacement of indigenous—often rare and endemic—species by generalist exotic species has been shown to promote biotic homogenisation at both regional and biogeographical scales, increasing species composition similarity among local assemblages [7–9]. Although native species can also drive biotic homogenisation in local assemblages (e.g., [10–12]), it is largely accepted that exotic species potentially drive biotic homogenisation in several groups of animals (e.g., [7, 9, 13–14]) and plants (e.g., [8, 11, 15–16]). On the other hand, exotic species can also select different habitats under specific ecological and environmental conditions [17], and thus lead to an increase in β-diversity [12, 18–19]. The identification of biotic homogenisation in island assemblages subjected to invasions can therefore help us to better understand the possible trajectories and effects of anthropogenic-driven invasions on indigenous assemblages [20–21].

In many cases, an increase in human-caused disturbances at the regional scale is the result of different rates and histories of local-scale disturbances, characterising a gradient in disturbance levels across sites. If there is a corresponding variation in species' tolerances to disturbance, less disturbed sites will harbour both disturbance-sensitive and disturbance-tolerant species, whereas the most disturbed sites will harbour only the most disturbance-tolerant species. In such a scenario, the process of biotic homogenisation is expected to lead to a nested pattern in species composition [22]. Nested patterns occur in biological communities when species-poor sites contain subsets of the assemblages found at species-rich sites, with the degree of nestedness quantifying the shared species composition between high- and low-diversity areas [22–24]. Nestedness is a pattern consisting of an ordered loss or gain of species across environmental or ecological gradients [25], which might contribute to faunal homogenisation on a regional scale—i.e., the predictable loss of species across assemblages while others are maintained may lead to more similar assemblages. However, the search for nestedness often leads to the discovery of other non-random patterns, termed as anti-nested [26–27]. An example of anti-nestedness would be when species-poor assemblages are mainly composed of species that are absent in richer assemblages. Under these circumstances, assemblages that are less nested than expected by chance usually exhibit dissimilarity patterns, such as species turnover and checkerboard distributions [27].

Nested and anti-nested patterns are typically explored using presence–absence matrices in which the entries represent the presence (1) or absence (0) of each species at each site [22, 28–29]. This approach in nestedness analyses allows changes in species composition to be detected through ordered gradients of species loss/gain between sites [30–33], thus providing information regarding assemblage structure and possible underlying factors [34]. Recently, presence-absence data have also been used as a powerful and sensitive tool to calculate the degree of assemblage homogenisation [11]. In our study, we used rarefaction on abundance data to obtain accurate and comparable presence-absence matrices enabling the quantification of biotic homogenisation.

The study of nestedness to assess biotic homogenisation on oceanic islands using different environmental variables as possible explanatory factors might be especially useful in understanding the dynamics of both extinction and diversification processes (mainly of indigenous species) and colonisation (mainly of exotic species). The Azores archipelago, in particular, has suffered a rapid and intensive conversion of native habitats [35]. As a consequence, currently about 58% of the arthropod species in the Azores Islands are thought to be exotics [36]. In this study, we explore the occurrence of nestedness/anti-nested patterns in Azorean epigean arthropods, in order to examine the role of habitat types on such patterns. Our aim is thus to investigate the current assembly patterns for the indigenous and exotic species assemblages of this archipelago as a probable consequence of differential extinction and colonisation processes, respectively. We hypothesised that i) indigenous species, which are often restricted to only some areas of native vegetation, would exhibit patterns less nested than those expected by chance; ii) on the contrary, exotic species, with putatively higher environmental tolerance and dispersal ability, would exhibit nestedness patterns due to selective colonisation of habitat types along a disturbance gradient; and, iii) the inclusion of exotic species into indigenous assemblages could increase the similarity of local assemblages in all habitat types (i.e. biotic homogenisation).

## Materials and Methods

### Study area

**Fig 1:**
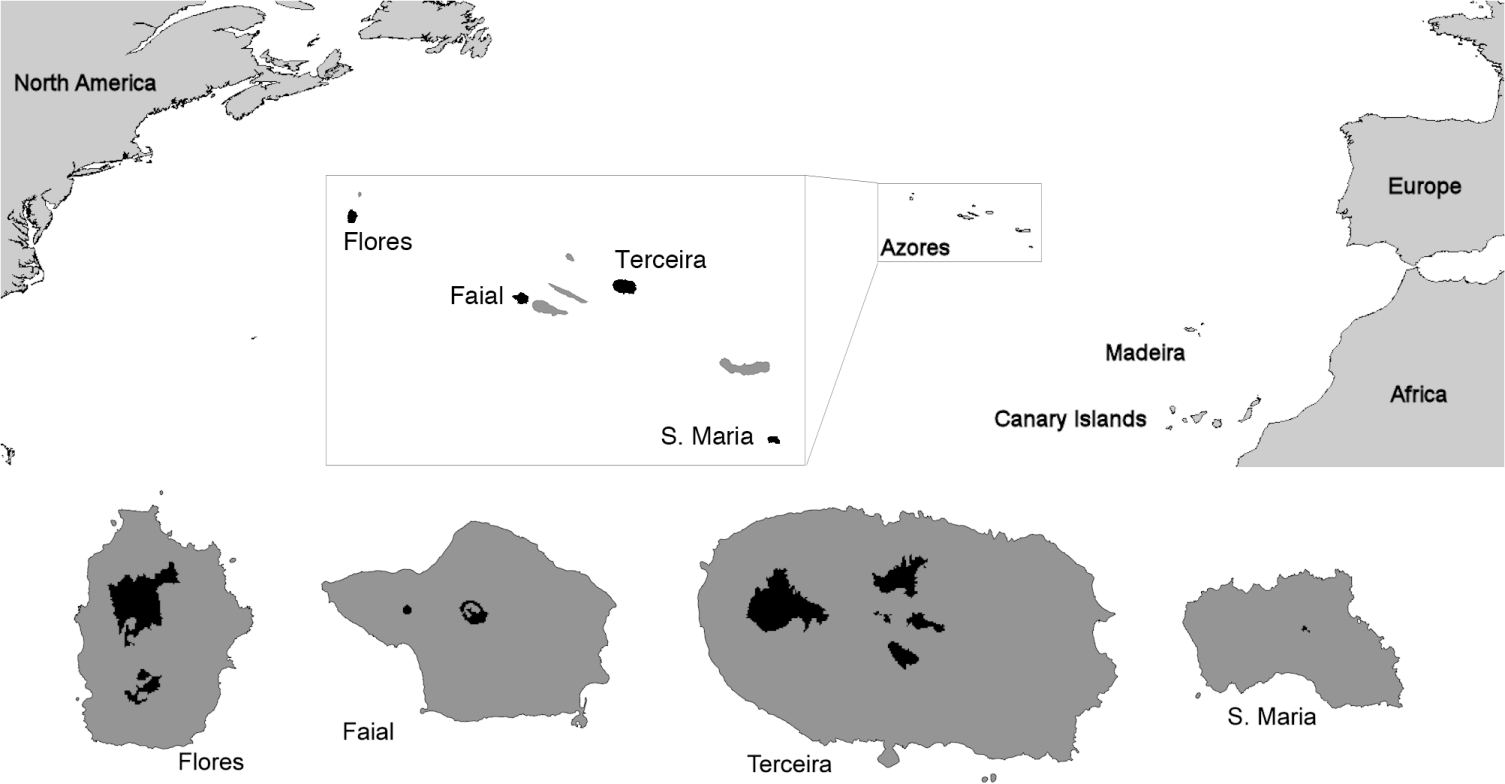
Location of the nine islands in the Azorean archipelago, aligned in a WNW–ESE direction. The study islands are highlighted (Flores, Faial, Terceira and Santa Maria), where a detail of the surface area occupied by native forests is also indicated in grey.

The Azores archipelago is located in the North Atlantic and comprises nine islands of volcanic origin (Fig 1), with a maximum age of 8.12 million years. The climate is temperate and oceanic, strongly influenced by both the surrounding ocean and island topography, with high levels of relative atmospheric humidity and limited temperature fluctuations throughout the year. The Azores were colonised close to six centuries ago, and the destruction of natural habitats was extensive. Current native forest cover represents only 2 to 5% of its original extent, which once encompassed almost the entire surface area of all the islands in the archipelago [35].

We considered four islands in this study, representing the range of environmental conditions, geographic locations and human disturbance levels found in the archipelago. The island of Santa Maria, located in the eastern part of the archipelago, is not only the driest island but also has the highest human population density (155 inhabitants per km^2^), the lowest percentage of native forest (0.1%) and the highest percentage of intensive pastures (44.8%). Two of the other considered islands (Terceira and Faial) are located in the central part of the archipelago. Terceira has the largest total area (400 km^2^) and native forest area (23.49 km^2^) of the four considered islands, whereas Faial is characterised by a low population density (22 inhabitants per km^2^) and a high proportion of semi-natural pastures (21.6%), with very few and small fragments of native forest (2.18 km^2^) (Fig 1). Finally, located in the western part of the archipelago, Flores harbours the highest percentage (but not area) of native forest (11.2%) of all the Azorean Islands [9].

### Sampling procedure

Four habitat categories, from the least to the most disturbed (see Fig A in S1 File), were considered: i) Native forest (*Laurisilva*), located at mid- to high altitudes and subjected to negligible degrees of human management, and dominated by the trees *Juniperus brevifolia*, *Laurus azorica*, *Ilex perado* subsp. *azorica*, and the shrubs *Vaccinium cylindraceum*, *Myrsine africana* and *Erica azorica*; ii) Exotic forest, located at all altitudes but more common at mid-elevations, consisting mostly of monospecific plantations of *Cryptomeria japonica*; iii) Semi-natural pastures, located at mid- to high altitudes, which are not intensively managed, with low grazing activity concentrated in spring and summer, and composed of native forbs (e.g. *Lotus uliginosus*), exotic and native grasses, rushes, sedges and ferns; iv) Intensively managed pastures, dominated by few exotic species (e.g. *Lolium perenne*, *Trifolim repens*), located at low altitudes and managed throughout the year. Permissions to conduct sampling procedures within the Azorean Natural Parks were obtained from the local authorities “Secretaria Regional da Educação, Ciência e Cultura, Direcção de Serviços de Ciência", and the "Direcção Regional do Ambiente", while permission to sample within private pastures was requested directly from the lands' owners. The Exotic forests of *C*. *japonica* are located in public lands managed by the Forest Services of the Azores, therefore sampling within these sites did not require authorisation. Our sampling protocol guaranteed the conservation of rare endemic species given our focus on small transects. The field studies did not involve endangered or protected species.

In the four considered islands, four transects were established in each of the four habitats (*n* = 4 × 4 × 4 = 64 transects). Each transect consisted of the placement of 30 pitfall traps spaced 5 m apart (see S1 File, for details). Traps were left in the field for two weeks, usually during the months of June, July or September of different years (native forests between 1997 and 2004, and the other habitats in 2008 and 2009, see S1 File). The possible effects of different sampling years on assemblage compositions were discarded after a between-year analysis using additional data [9]. All Araneae, Opiliones, Pseudoscorpiones, Diplopoda, Chilopoda and Insects (excluding Collembola, Diplura, Diptera and Hymenoptera) were identified (see S1 File, for details).

### Preliminary data analysis

Arthropods were grouped into two colonisation categories: indigenous (129 species) and exotics (160 species). Indigenous species are those occurring only in the Azores (endemics) and/or those present in the nearby Madeira and Canary archipelagos and/or continental areas (indigenous non-endemics). The non-endemic indigenous species supposedly arrived at the Azorean archipelago via natural long-distance dispersal mechanisms before historical times. Exotic species are those introduced outside their native distribution and are believed to be in the archipelago due to recent human activities being usually accidentally introduced. Approximately 16% of taxa were left unidentified at species level and these were classified within the same colonisation category as that of the other taxa belonging to the same genera, subfamilies or families, when all these taxa belonged to the same category (according to [36]). The remaining species were assumed to be indigenous, as exotics are usually widespread and easier to identify (see Table A in S1 File, for detailed colonisation categories).

**Fig 2:**
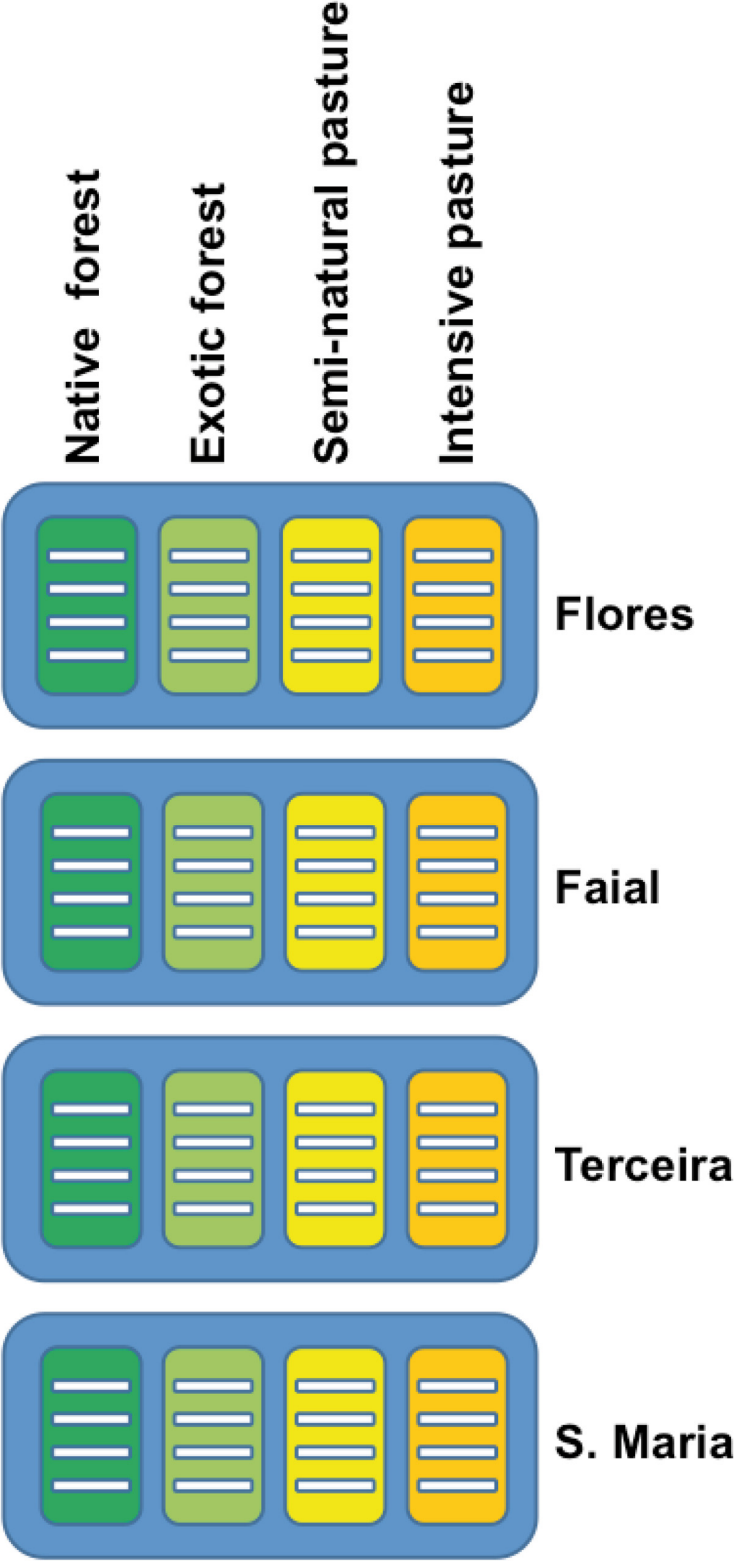
Schematic representation of the sampling procedure. Each line indicates each sampled transect, the blue boxes represent the four islands (Flores, Faial, Terceira and Santa Maria), and the colour boxes inside of each island represent each habitat (Native forest, Exotic forest, Semi-natural pasture, and Intensive pasture). We compared all transects at the archipelago level (four islands and four habitats together, n = 64 transects), and separately within each island alone (n = 16 transects), and also within each habitat considering the four study islands together (n = 16 transects).

The abundance values for each species obtained in the 30 pitfall traps of each transect were pooled to construct a primary matrix for all 64 transects. As the survey effort required to reach reliable inventories for each transect differed depending on habitat category [9], we built comparable abundance-based rarefied matrices, selecting a number of individuals at random in each transect, equal to the minimum number obtained in any of the considered transects (62 individuals). We repeated this process 10 times. These abundance matrices were transformed into presence–absence matrices to estimate the degree of nestedness and β-diversity values independently of differences in abundance. From these matrices, independent matrices for each one of the four considered habitats and each one of the four studied islands were built considering both indigenous and exotic species (see S1 File, for details of the abundance-based rarefaction process). Thus, we examine all the transect data at the archipelago level (four islands and four habitats together, n = 64 transects), but also the data coming from each island independently of the habitat (n = 16 transects) or coming from each habitat independently of the island (n = 16 transects, Fig 2).

### Nestedness analyses

The detection of nestedness in presence–absence matrices depends on the metric used to quantify the degree of nestedness [22, 29]. Here, we used the NODF index given its relative independence of matrix size and shape [28]. The NODF values range from 0 to 100, corresponding to minimum and maximum degrees of nestedness, respectively. To evaluate the extent to which the overall degree of nestedness is determined by differences in species composition among habitats, or by differences in species occurrence along the species richness gradient, we also calculated nestedness only among columns (transects, NODF_transects_) and only among rows (species, NODF_species_), respectively [25, 37]. Therefore, we obtained three measures: NODF (overall nestedness), NODF_transects_, and NODF_species_. Two common circumstances may influence the calculation of NODF values: species distributions and sampling effort. The widespread distribution of abundant species in contrast to the narrow distribution of rare species can increase the degree of nestedness through a passive sampling effect [22, 38–39]. Similarly, the use of different sampling efforts per site can potentially increase the degree of nestedness [40–41]. In this study we compared the values of NODF calculated on the raw matrices with those calculated on rarefied matrices because the two formerly mentioned potential artefacts might increase the overall degree of nestedness when calculated on raw data. We used the NODF program (Version 2.0 available at www.umk.pl/~ulrichw) for all these calculations [37].

The detection of nestedness is dependent upon the selected null model to calculate a significant departure from a random species distribution [22, 29]. An examination of the results provided by different null models allows disentangling the possible mechanisms driving the observed species patterns [42]. To properly detect nested/anti-nested patterns and better understand the underlying mechanisms, we used a combination of five different null models (see S2 File). For simplicity, we present here only the four-step proportional–proportional (PP) algorithm, which is considered a restrictive null model with sufficient power to discriminate nested from anti-nested patterns [43]. In this model, presences per row (species) and column (transects) vary randomly, but the mean row and column totals are fixed to those of the original matrix. This null model is considered the most realistic and mimics random colonisations in a metacommunity of small-scale surveys in which occurrences are expected to vary substantially [43]. We used both the 5th and 95th percentiles of the NODF values generated by the null models to discriminate between significant nestedness (above the 95th percentile) and significant anti-nestedness (below the 5th percentile).

### Biotic homogenisation

In order to assess whether the incorporation of exotic species into indigenous assemblages increases or reduces the assemblage similarity across habitats, we calculated the mean pairwise dissimilarity (β-diversity) between the local assemblages of each habitat considering the four study islands together (Fig 2). We used a modification (see [44]) of the species replacement index proposed by Williams [45], whose values are independent of species richness differences:

$$\beta =2\frac{\text{min}(b,c)}{a+b+c};$$

where *a* indicates the species co-occurring in two different sites, and *b* and *c* are the exclusive species occurring only in each one of the two sites.

We calculated the species replacement index for each pairwise comparison between transects (n = 120), using the presence-absence data extracted from the 10 rarefied matrices and performing the analyses in the ‘BAT’ package [46] of R software 2.14.2 [47]. These analyses were performed for the whole assemblages (i.e. containing both indigenous and exotic species) and separately for the indigenous species alone. All these pairwise values (n = 120) were compared between the whole and the indigenous assemblages by a non-parametric Wilcoxon matched pair test to estimate if the inclusion of exotic species into indigenous assemblages increased/decreased the β-diversity values.

## Results

**Fig 3:**
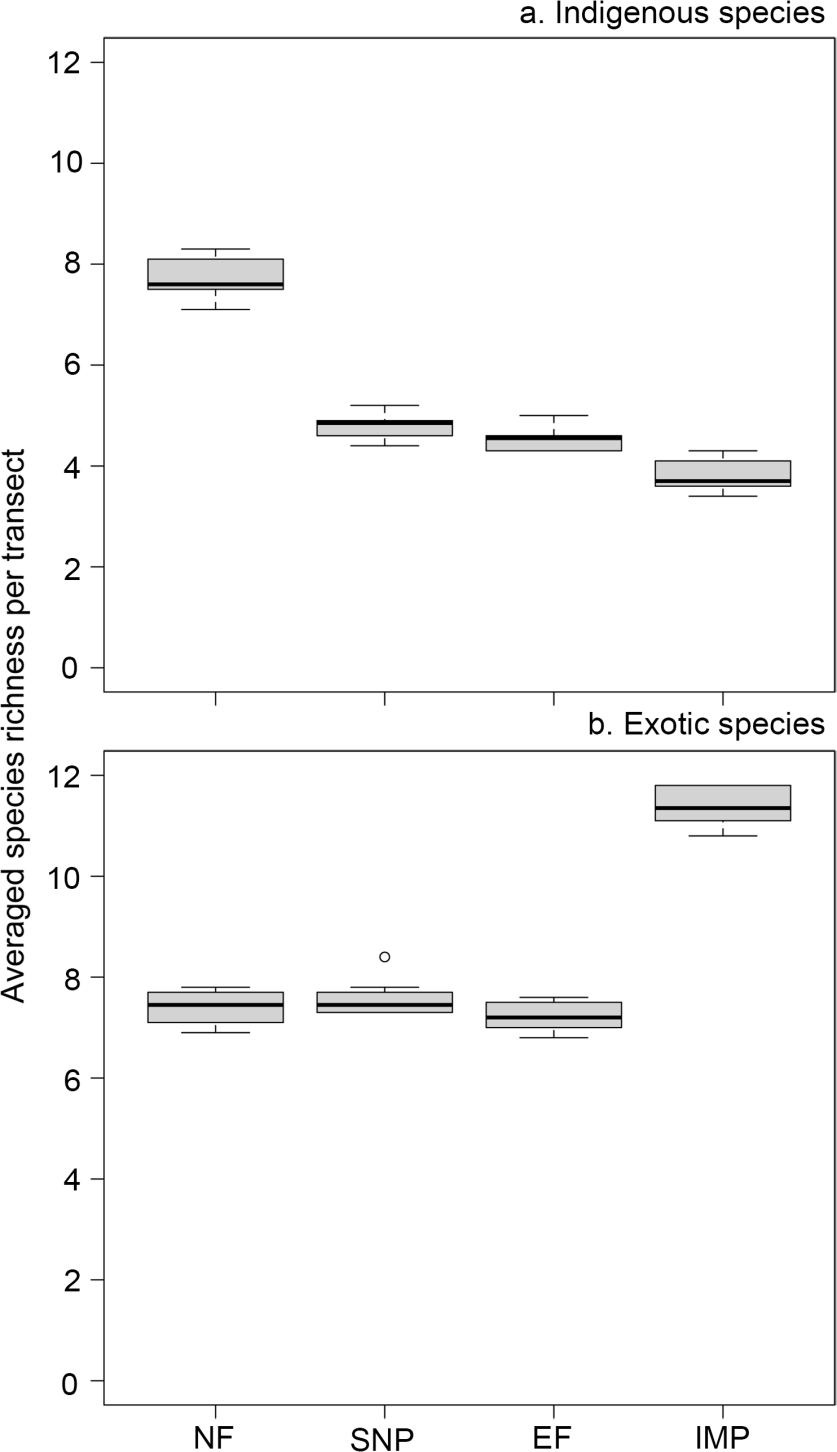
Averaged species richness per transect for each habitat type. Box plot indicates the median, maximum, minimum, and upper and lower quartiles of the averaged species richness per transect calculated per each considered habitat: Native forest (NF), Semi-natural pastures (SNP), Exotic forest (EF), and Intensively managed pasture (IMP). Species richness of (a) indigenous and (b) exotic species were calculated using the 10 rarefied matrices.

We found opposing responses of the indigenous and exotic arthropods to the disturbance gradient (Fig 3). The species richness of indigenous arthropods decreased from native forests to intensively managed pastures (Fig 3A), whereas the species richness of exotic arthropods had a positive relationship with disturbance level (Fig 3B).

Although different nested (random and anti-nested) patterns were detected the values of NODF_transects_ were always higher than those of NODF_species_ showing the same pattern for the whole assemblage and separately for indigenous and exotic species (see Table B in S3 File).

### Between-island differences

The degree of nestedness calculated independently for each island revealed assemblages exhibiting random and significant anti-nested patterns whereas nested patterns were not detected (Table 1). Notably, these opposite patterns were detected on both indigenous and exotic assemblages. Particularly, we detected significant anti-nested patterns in Santa Maria and Faial for indigenous species and in Santa Maria for exotic species (Table 1).

**Table 1:** Degree of nestedness independently calculated on each study island, indicating significant anti-nestedness.

Species origin	Island	Means NODF_rarefied_	SD_rarefied_	P > 0.95
**Indigenous species**				
	**Flores**	26.34	2.32	0
	**Terceira**	19.75	2.18	0
	**Santa Maria**	18.59	2.96	2
	**Faial**	21.18	2.50	8
**Exotic species**				
	**Flores**	30.50	2.11	0
	**Terceira**	18.87	0.78	0
	**Santa Maria**	24.55	1.07	8
	**Faial**	26.76	1.97	0

Presence-absence data of the ten rarefied matrices were considered separately for indigenous and exotic species. NODF_rarefied_ indicates the mean degree of nestedness (SD values are also indicated). The PP null model (PP is the proportional–proportional null model) was applied. The final column, labelled P > 0.95, indicates the number of rarefied matrices out of 10 that showed statistically significant anti-nestedness (see Table D in S3 File, for detailed values on rarefied matrices and Table E in S3 File, for the raw data). All the significant NODF values indicate a lower degree of nestedness than those obtained in all the 1000 null model permutations (P > 0.999; P > 0.95), indicating anti-nestedness.

### The effect of habitat

In both indigenous and exotic assemblages, the four considered habitats always showed random and anti-nested patterns (Table 2). For indigenous species, anti-nested patterns occurred only in native forest, whereas intensive pastures and exotic forest showed significant anti-nestedness patterns in the case of exotic species (Table 2). The addition of exotic species to the indigenous assemblages of native forest (whole assemblages) did not change the observed anti-nested patterns of local assemblages. On the contrary, the inclusion of exotic species even increased the number of significant anti-nested patterns in the most disturbed habitats (intensive pastures, exotic forests and semi-natural pastures; Table 2). Particularly, the addition of exotic species to the indigenous assemblages increased overall species dissimilarity (Table 3), such increase being especially relevant in intensive and semi-natural pastures. Only in four cases, the inclusion of exotic species in native forests reduced the species replacement, indicating possible assemblage homogenisation (Table 3). In all the analyses, we always detected that NODF values calculated on rarefied matrices were lower than those calculated on raw matrices (see Table B, Table E and Table G in S3 File).

**Table 2:** Degree of nestedness calculated on each habitat considering the four study islands together, indicating significant anti-nestedness.

Species origin	Habitats	Means NODF_rarefied_	SD_rarefied_	P > 0.95
**Whole assemblages**				
	**Native forest**	23.71	1.10	4
	**Semi-natural pasture**	24.01	1.11	3
	**Exotic forest**	29.33	1.67	4
	**Intensive pasture**	23.34	1.31	5
**Indigenous species**				
	**Native forest**	22.94	2.37	5
	**Semi-natural pasture**	25.59	2.85	0
	**Exotic forest**	23.27	2.00	0
	**Intensive pasture**	20.18	2.24	0
**Exotics species**				
	**Native forest**	27.39	2.38	1
	**Semi-natural pastures**	26.1	1.27	1
	**Exotic forest**	38.58	3.79	3
	**Intensive pastures**	25.49	1.42	4

Presence-absence data of the ten rarefied matrices were considered for native forest, semi-natural pasture, exotic forest, and intensive pasture, and separately for the whole assemblages, and for indigenous and exotic species. NODF_rarefied_ indicates the mean degree of nestedness (SD values are also indicated). The PP null model (PP is the proportional–proportional null model) was applied. The final column, labelled P > 0.95, indicates the number of rarefied matrices out of 10 that showed statistically significant anti-nestedness (see Table F in S3 File, for detailed values on rarefied matrices and Table G in S3 File, for the raw data). All the significant NODF values indicate a lower degree of nestedness than those obtained in all the 1,000 null model permutations (P > 0.999; P > 0.95), indicating anti-nestedness.

**Table 3:** General increase in assemblage dissimilarity by the inclusion of exotic species into indigenous assemblages.

	Native forest		Semi-natural pasture		Exotic forest		Intensive pasture	
	Means β	SD	Means β	SD	Means β	SD	Means β	SD
β **All**	0.65	0.03	0.64	0.02	0.57	0.03	0.65	0.03
β **Indigenous**	0.66	0.04	0.58	0.05	0.53	0.04	0.58	0.05
**Wilcoxon Z values**	2.17	1.29	2.87	1.58	1.39	1.44	2.99	1.39
**Nc dissimilarity**	1		8		1		8	
**Nc homogenisation**	4		0		0		0	

β indicates the averaged values of the species replacement index calculated per each considered habitat category and for the whole epigean arthropod assemblages (All), and indigenous species alone. Averaged Wilcoxon Z values for the 10 rarefied matrices and numbers of cases (Nc) in which the similarity values of the whole assemblages and the indigenous species alone are statistically different (*P* < 0.05, SD is indicated for the β and Z values) are indicated. All cases of significant differences indicate that the inclusion of exotic species into the indigenous assemblages (All) increased the dissimilarity of the whole assemblages, with the exception of four cases in native forests indicating an increase in assemblage similarity or biotic homogenisation (see Table H in S3 File, for the Z values of the ten rarefied matrices).

## Discussion

We detected significant anti-nested patterns in the epigean ground dwelling arthropod assemblages, suggesting a lack of biotic homogenisation. Furthermore, we did not detect nestedness when islands or habitats were analysed separately. Our results thus indicate that these assemblages do not seem to exhibit a gradual loss of species following a nested structure within islands or habitats (see [42]). Therefore, both indigenous and exotic species could establish singular and idiosyncratic local assemblages on different islands or in different habitats. These results confirm our first hypothesis on obtaining patterns less nested than expected by chance in indigenous assemblages; but, contrary to our expectations, in many cases exotic species were also less nested than would have been expected by chance and did not seem to promote biotic homogenisation of local assemblages. Why did we not observe the expected homogeneity in the assemblages with the invasion of exotic species? Following the first human settlement six centuries ago, rapid changes in the original vegetation have promoted a drastic reduction in the original native forest areas of the Azores Islands [35]. Historical extinctions associated with these former changes probably favoured a more generalist widespread fauna of indigenous species across habitats [9, 35, 48], thus diminishing our benchmark dissimilarity among local assemblages. In our study, the proportion of indigenous species that occurred exclusively in native forests was higher for endemic (33%) than for non-endemic (12%) species; we thus suggest that historical extinctions involving the most specialised endemics have taken place in the disturbed habitats. The spatial and temporal extinction of specialists in favour of other indigenous generalists has often promoted biotic homogenisation under anthropogenic perturbations—for example, under habitat homogenisation, environmental changes or the expansion of urban areas [10, 14–15]. Thus the inclusion of exotic species does not have to trigger homogenisation, for example, when we consider different scales of observation. While the introduction of widespread exotics in distant indigenous assemblages can drive biotic homogenisation if analysed at a regional scale [49], our results and other studies (see [12,19]) indicate that these introductions seem to increase local assemblage heterogeneity.

Previous results using abundance data for the same archipelago [9] showed that the inclusion of exotic species generally increased the similarity of local assemblages by 21% in the case of natural forests, and 55% for intensive pasturelands. The present results indicate that such faunal homogenisation is not apparent when incidence data are used. Therefore, less abundant and probably more specialised exotic species seem to colonise some local assemblages and not others, promoting their compositional heterogeneity. Although the most abundant exotic species are the same everywhere (e.g., *Oedothorax fuscus* in semi-natural and intensively managed pastures, or *Blaniulus guttulatus* in native and exotic forests [9]), the rarest are subject to constant replacement in both space and time, hence causing large differences in incidence data, but having little influence on indices of assemblage similarity. Although the measurement of biotic homogenisation has been largely based on abundance data [18, 50], the use of an index accounting for abundances instead of incidences seems to be less sensitive under changes associated with gain/loss of species [11]. In our study, rare species are considered independent of differences in sampling efforts, revealing that the species replacement observed with the incidence data indicates reliable differences. Therefore, we recommend the use of abundance-based rarefied matrices converted to incidence data rather than using raw data, to diminish the effect of abundant species and of differences in sampling effort for the detection of nested or anti-nested patterns. Although differences in species abundances and sampling efforts have been traditionally considered for nestedness estimations, few studies have suggested solutions based, for example, on considering sampling bias [40] or individual-based models [41]. Rare species, often tourists, or belonging to sink populations, may have a large influence on nestedness analyses [51]. Rarefaction is a possible solution to avoid this problem [52–54]. In our study, this procedure resulted in lower nestedness values than those generated by the use of raw data, thus increasing the chance of obtaining anti-nested patterns.

Finding biotic homogenisation depends on the spatial and temporal scale at which assemblages are compared [49–50, 55], the starting frequencies of exotics and natives and the length of the gradient over which homogenisation is measured [12, 55]. Although we had no data for assessing temporal homogenisation in our study, we argue that current indigenous assemblages are the consequence of a historical trajectory of extinctions. Such historical extinctions together with the differential introduction of novel exotic species in the disturbed habitats seem to have promoted the construction of the new assemblages detected in human-altered landscapes. Notably, and contrary to expectations, our study suggests that the assemblages of exotics are clearly environmentally structured. Furthermore, the inclusion of exotic species in the original indigenous-only assemblages does not significantly increase the compositional homogeneity of the local assemblages present in the native forests, but rather generates additional anti-nested patterns in the most disturbed habitats (i.e., intensive pastures, exotic forests, and even in the less degraded semi-natural pastures), even revealing an increase in the turnover of species in these habitats. Although invaders are usually considered as opportunistic generalists, they might also be habitat specialists leading to idiosyncratic invasion processes [56–57]. The current literature concerning the consequences of the integration of exotic insect species into indigenous assemblages inhabiting oceanic islands suggests that invaders might, on many occasions, be as specialised as indigenous species [58]. The small size of interaction networks [59] and the presence of native super-generalist species on oceanic islands [60] might facilitate this integration of exotic species into local assemblages. Our results suggest that such invasions can also generate new local assemblages within degraded habitats. The inclusion of exotic species within assemblages of human-altered habitats might play an important role in ecosystem functioning and resilience, replacing functions that otherwise would be lost because of the local extinction of most intolerant indigenous species [61–62]. Therefore, the replacement of indigenous species by exotics with similar functional traits might result in small changes in ecosystem functioning [63]. This assertion might somehow relate to the lack of common functional traits shared by invaders in the literature (e.g., [56–57, 64]) and their heterogeneous and context-dependent impacts on native assemblages [65]. In our case, we expected the homogenisation of the local assemblages located in native forests due to the incorporation of exotic species. However, our results show that the inclusion of a considerable number of exotic species (about 12 species on average) within indigenous local assemblages (about 13 species on average) does not appear to diminish their degree of idiosyncrasy in the native forests. Previous studies suggest the existence of a facilitation process in which introduced species might serve as a resource for generalist native species [66], mainly predators. Thus, exotic species can play different roles in native assemblages, according to their trophic position. If these biotic interactions involve the occurrence of target species, the colonisation of exotic species would not necessarily lead to a faunistic homogenisation. For instance, tight associations between exotic predators, feeding only on specific preys, would lead to more heterogeneous assemblages through the selective disappearance of prey species, in comparison with those non-colonised habitats by exotic species. Previous results obtained in the Azores archipelago suggest that exotic species not only replace, but mainly increase the functional space of the assemblages of these islands [67], which may be conditioned by the trajectory of the current assemblages that have undergone an intense and prolonged history of human alterations. Further studies are necessary to estimate the functional role played by these exotic species within the current native forests, as well as their relevance in ecosystem processes.

## Supporting Information

S1 File Calculation of the landscape disturbance index to assess habitat disturbance (Fig A), sampling details across transects and taxonomical identifications, process of abundance-based rarefaction, and taxonomic list indicating the colonisation category (indigenous or exotic) of the species (Table A). (PDF)

S2 File Description of the five null models used to determine the significance of nested/anti-nested patterns on raw data and rarefied matrices; we selected from least to most restrictive null models in detecting nested patterns. (PDF)

S3 File Degree of nestedness (NODF) calculated on the raw data and on the ten rarefied matrices (1–10) using presence–absence data of the whole assemblages, and of both indigenous and exotic species separately, for the considered regional data (Table B and Table C) and also separately for the four study islands (Table D and Table E on rarefied and raw data, respectively) and the four study habitats (Table F and Table G, on rarefied and raw data, respectively). An index of species replacement was also calculated to compare the β-diversity of whole assemblages (including indigenous and exotic species) and of the indigenous species alone (Table H).
(PDF)

S4 File The ten rarefied matrices used for this study. (PDF)
