# Supplementary material for: The Colonisation of Exotic Species Does Not Have to Trigger Faunal Homogenisation: Lessons from the Assembly Patterns of Arthropods on Oceanic Islands
Source: PLoS One. 2015 May 29;10(5):e0128276. doi: 10.1371/journal.pone.0128276 (PMC4449220; doi:10.1371/journal.pone.0128276)
Supplement: S1 File — (PDF) [file pone.0128276.s001.pdf]

S1 File. Calculation of the landscape disturbance index to assess habitat disturbance (Fig. A), sampling details across transects and taxonomical identifications, process of abundance-based rarefaction, and taxonomic list indicating the colonisation category (indigenous or exotic) of the species (Table A).

Habitat disturbance

We calculated an index of landscape disturbance ranging from 0 to 100 [1] to represent local habitat disturbance (Fig. A). This index of disturbance increased from the least (native forests) to the most disturbed habitat (intensively managed pastures) considered in the study.

The index of landscape disturbance was calculated as follow [1]. First, we considered different anthropogenic landscape alterations with a different value of "local disturbance" (L): native forests = 0, naturalized vegetation (i.e. usually abandoned pasturelands with spontaneous growth of exotic and native plants) = 1, exotic forest = 2, semi-natural pasture = 3; intensive pasture = 4; orchards = 5; urban/industrial = 6. The sea is equal to "no data". The landscape disturbance of each 100 * 100m grid section in the island was calculated as:

$$D_{i,j} = \left(\frac{2L_{i,j} + \sum_{n=1}^{r}\sum_{m=1}^{c}\frac{L_{n,m}}{d_{(i,j)(n,m)^2}}}{2\max + \sum_{n=1}^{r}\sum_{m=1}^{c}\frac{\max}{d_{(i,j)(n,m)^2}}} \right) \times 100$$

where: *Di,j* is landscape disturbance of the cell (ranged from 0 to 100); *L* is local disturbance of each cell; *r* is number of rows in the map; *c* is number of columns in the map; *d* is distance between two cells; max is maximum theoretical value of disturbance

each cell may take (in this case max = 6). Thus, the influence of each cell surrounding the focal cell is inversely proportional to the square of the distance between the two cells. That is, a cell next to the focal cell (d = 1) has 4 times more influence that a cell two rows apart (d = 2). Although all cells in the island were taken into account, the ones far away from the focal cell had an almost negligible individual influence. The *L* value of the focal cell was multiplied by 2 to guarantee that the landscape alteration of the focal cell ($d = 0$) was double weighted in comparison with the immediately surrounding cells ($d = 1$). Also, the division by the maximum value of each cell was necessary to guarantee that the presence of the ocean would not make all coastal cells to have low *D* (as if the ocean was equivalent to native forest ($L = 0$)). As a beneficial side-effect, this division by the theoretical maximum also guarantees that *D* is not open-scaled since *D* must always be between 0 (no disturbance at all, only possible if all cells had native forest) and 100 (maximum possible disturbance, only possible if all cells had urban/industrial).

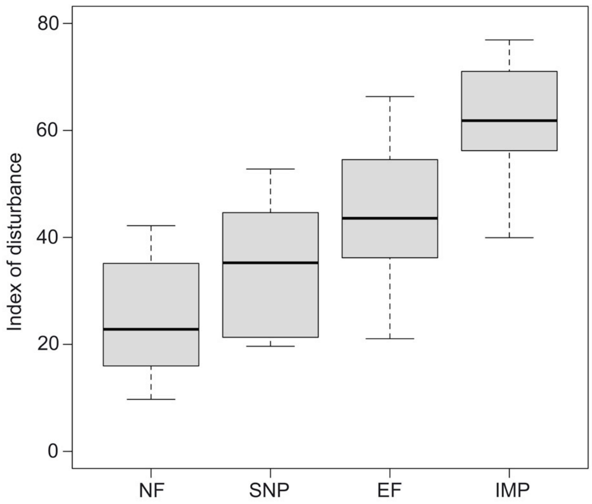

Fig. A. Box plot indicating the median, maximum, minimum, and upper and lower quartiles of the index of disturbance in the four considered habitats: Native forest (NF), Semi-natural pastures (SNP), Exotic forest (EF) and Intensively managed pasture (IMP).

Sampling details across transects and taxonomical identifications

Four transects (sampling units) were randomly selected per each combination of habitat and island (n = 64). Native forests were sampled between 1997 and 2004, always during summer (Borges et al., 2005 [2]). Data were collected in the other three habitats on Santa Maria, Faial and Flores during the summer of 2009 (see also Meijer et al., 2011 [3]) and on Terceira during the summer of 2008 (see Cardoso et al., 2009 [4]).

Each 150 m transect consisted of 30 pitfall traps spaced 5 m apart. The pitfall traps were plastic cups with diameters of 42 mm and depths of 78 mm; they were buried in the ground so that the rims of the cups were level with the surface. Half of the traps in each transect were filled with approximately 60 ml of an antifreeze liquid (diluted ethylene glycol), and the other half were filled with the same volume of an attractive Turquin solution made of dark beer, and for each litre of beer, 10 g of chloral hydrate, 5 ml formalin and 5ml glacial acetic acid. The ethylene and Turquin traps were alternated in

each transect. Traps were left in the field for 2 weeks. The obtained arthropods were preserved at 70% ethanol for identification in the laboratory at the species level for the taxa Araneae, Opiliones, Pseudoscorpiones, Diplopoda, Chilopoda and Insects (excluding Collembola, Diplura, Diptera and Hymenoptera). Taxonomic identification was performed in two steps: 1) trained parataxonomists sorted samples into morphospecies (or RTUs, i.e. recognizable taxonomic units, *sensu* [5]) using a non-complete reference collection; 2) experienced taxonomists assisted in the identification of the morphospecies: Andrew Polaszek, António Bivar Sousa, Artur Serrano, Arturo Baz, Fernando Ilharco, Henrik Enghoff, Jordi Ribes, José Quartau, Jörg Wunderlich, Mário Boieiro, Ole Karsholt, Richard zur Strassen, Volker Assing, Volker Manhert and Virgílio Vieira. Because approximately only 16% of taxa were left unidentified, we use the term 'species' throughout the text for simplicity.

Abundance-based rarefaction

In order to obtain comparable matrices of species compositions independently of possible differences in sampling efforts, we constructed abundance-based rarefied matrices using a matrix containing both exotic and indigenous species.

1) We selected a number of individuals at random in each transect equal to the minimum number obtained in any of the considered transects (62 individuals). This process was performed using the following line command in the R software ([6]; 'vegan' package, [7]), where 'comm' is the original data matrix:

   rrarefy(comm, min(rowSums(comm)))

2) This process was repeated 10 times.
3) The obtained abundance matrices were subsequently transformed into presence–absence matrices.
4) These presence-absence matrices were used to separately generate matrices

representing the data belonging to each considered habitat (independently of the island) and to each studied island (independently of the habitat), considering both indigenous and exotic species separately.

Table A. Taxonomic list indicating the assumed colonisation category (exotic Ex or indigenous Ind) of the species (* indicates endemic species for the indigenous category).

**Order**	**Family**	**Species**	Ex/Ind
Araneae	Agelenidae	*Tegenaria domestica* (Clerck)	Ex
Araneae	Clubionidae	*Clubiona terrestri*s Westring	Ex
Araneae	Dysderidae	*Dysdera crocata* C. L. Koch	Ex
Araneae	Gnaphosidae	*Zelotes aeneus* (Simon)	Ex
Araneae	Gnaphosidae	*Leptodrassus albidus* Simon	Ex
Araneae	Gnaphosidae	*Trachyzelotes lyonneti* (Audouin)	Ex
Araneae	Gnaphosidae	*Micaria pallipes* (Lucas)	Ex
Araneae	Gnaphosidae	*Haplodrassus signifer* (C.L. Koch)	Ex
Araneae	Gnaphosidae	*Zelotes tenuis* (L. Koch)	Ex
Araneae	Linyphiidae	*Oedothorax fuscus* (Blackwall)	Ex
Araneae	Linyphiidae	*Tenuiphantes tenuis* (Blackwall)	Ex
Araneae	Linyphiidae	*Erigone autumnalis* Emerton	Ex
Araneae	Linyphiidae	*Erigone dentipalpis* (Wider)	Ex
Araneae	Linyphiidae	*Meioneta fuscipalpa* (C.L. Koch)	Ex
Araneae	Linyphiidae	*Mermessus fradeorum* (Berland)	Ex
Araneae	Linyphiidae	*Ostearius melanopygius* (O.P.-Cambridge)	Ex
Araneae	Linyphiidae	*Prinerigone vagans* (Audouin)	Ex
Araneae	Linyphiidae	*Erigone atra* Blackwall	Ex
Araneae	Linyphiidae	*Pelecopsis parallela* (Wider)	Ex
Araneae	Linyphiidae	*Agyneta decora* (O.P.-Cambridge)	Ex
Araneae	Linyphiidae	*Mermessus bryantae* (Ivie & Barrows)	Ex
Araneae	Linyphiidae	*Erigone* sp.1	Ex
Araneae	Linyphiidae	*Microlinyphia johnsoni* (Blackwall)	Ex
Araneae	Mimetidae	*Ero furcata* (Villers)	Ex
Araneae	Oecobiidae	*Oecobius navus* Blackwall	Ex
Araneae	Salticidae	*Chalcoscirtus infimus* (Simon)	Ex
Araneae	Salticidae	*Heliophanus kochii* Simon	Ex
Araneae	Salticidae	Gen. sp.1	Ex
Araneae	Tetragnathidae	*Pachygnatha degeeri* Sundevall	Ex
Araneae	Tetragnathidae	*Metellina merianae* (Scopoli)	Ex
Araneae	Theridiidae	*Cryptachaea blattea* (Urquhart)	Ex
Araneae	Theridiidae	*Steatoda grossa* (C.L. Koch)	Ex
Araneae	Thomisidae	*Xysticus nubilus* Simon	Ex
Araneae	Zodariidae	*Zodarion atlanticum* Pekár & Cardoso	Ex
Araneae	Araneidae	*Gibbaranea occidentalis* Wunderlich	Ind*
Araneae	Linyphiidae	*Meioneta depigmentata* (Wunderlich)	Ind*

Araneae	Linyphiidae	*Canariphantes acoreensis* (Wunderlich)	Ind*
Araneae	Linyphiidae	*Canariphantes relictus* Crespo & Bosmans	Ind*
Araneae	Linyphiidae	*Acorigone acoreensis* (Wunderlich)	Ind*
Araneae	Linyphiidae	*Minicia floresensis* Wunderlich	Ind*
Araneae	Linyphiidae	*Porrhomma borgesi* Wunderlich	Ind*
Araneae	Linyphiidae	*Canariphantes junipericola* Crespo & Bosmans	Ind*
Araneae	Lycosidae	*Pardosa acorensis* Simon	Ind*
Araneae	Pisauridae	*Pisaura acoreensis* Wunderlich	Ind*
Araneae	Salticidae	*Neon acoreensis* Wunderlich	Ind*
Araneae	Theridiidae	*Rugathodes acoreensis* Wunderlich	Ind*
Araneae	Dictynidae	*Lathys dentichelis* (Simon)	Ind
Araneae	Linyphiidae	*Tenuiphantes miguelensis* Wunderlich	Ind
Araneae	Linyphiidae	*Palliduphantes schmitzi* (Kulczynski)	Ind
Araneae	Oecobiidae	*Oecobius similis* Kulczynski	Ind
Araneae	Theridiidae	*Theridion musivivum* Schmidt	Ind
Araneae	Thomisidae	*Xysticus cor* Canestrini	Ind
Blattaria	Blattellidae	*Loboptera decipiens* (Germar)	Ind
Blattaria	Polyphagidae	*Zetha vestita* (Brullé)	Ind
Chordeumatida	Haplobainosomatidae	*Haplobainosoma lusitanum* Verhoeff	Ex
Coleoptera	Phalacridae	Gen. sp.2	Ex
Coleoptera	Anthicidae	Gen. sp.2	Ex
Coleoptera	Anthicidae	Gen. sp.1	Ex
Coleoptera	Bothrideridae	*Anommatus duodecimstriatus* (Muller)	Ex
Coleoptera	Carabidae	*Paranchus albipes* (Fabricius)	Ex
Coleoptera	Carabidae	*Anisodactylus binotatus* (Fabricius)	Ex
Coleoptera	Carabidae	*Pterostichus vernalis* (Panzer)	Ex
Coleoptera	Carabidae	*Pseudophonus rufipes* (DeGeer)	Ex
Coleoptera	Carabidae	*Amara aenea* (De Geer)	Ex
Coleoptera	Carabidae	*Harpalus distinguendus* (Duftschmidt)	Ex
Coleoptera	Carabidae	*Agonum muelleri* (Herbst)	Ex
Coleoptera	Carabidae	*Laemosthenes complanatus* Dejean	Ex
Coleoptera	Chrysomelidae	*Chaetocnema hortensis* (Fourcroy)	Ex
Coleoptera	Chrysomelidae	*Longitarsus kutscherae* (Rye)	Ex
Coleoptera	Chrysomelidae	*Bruchus* sp.2	Ex
Coleoptera	Chrysomelidae	*Psylliodes chrysocephalus* (Linnaeus)	Ex
Coleoptera	Chrysomelidae	*Epitrix cucumeris* Harris	Ex

Coleoptera	Coccinellidae	*Nephus helgae* Fursh	Ex
Coleoptera	Corylophidae	*Sericoderus lateralis* (Gyllenhal)	Ex
Coleoptera	Corylophidae	Gen. sp.5	Ex
Coleoptera	Cryptophagidae	*Cryptophagus* sp.1	Ex
Coleoptera	Cryptophagidae	*Cryptophagus* sp.9	Ex
Coleoptera	Cryptophagidae	*Cryptophagus* sp.2	Ex
Coleoptera	Cryptophagidae	*Cryptophagus* sp.3	Ex
Coleoptera	Cryptophagidae	*Cryptophagus* cf. *cellaris*	Ex
Coleoptera	Curculionidae	*Sitona discoideus* Gyllenhal	Ex
Coleoptera	Curculionidae	*Otiorhynchus cribicollis* Gyllenhal	Ex
Coleoptera	Curculionidae	*Coccotrypes carpophagus* (Hornung)	Ex
Coleoptera	Curculionidae	*Sitona* sp.1	Ex
Coleoptera	Curculionidae	*Otiorhynchus rugosostriatus* (Goeze)	Ex
Coleoptera	Curculionidae	*Xyleborinus alni* Nijima	Ex
Coleoptera	Curculionidae	*Sitona* sp.5	Ex
Coleoptera	Curculionidae	*Gymnetron pascuorum* (Gyllenhal)	Ex
Coleoptera	Curculionidae	*Sitona puberulus* Reitter	Ex
Coleoptera	Curculionidae	*Pantomorus cervinus* (Boheman)	Ex
Coleoptera	Curculionidae	Gen. sp.1	Ex
Coleoptera	Curculionidae	*Tychius picirostris* (Fabricius)	Ex
Coleoptera	Curculionidae	*Sitona* sp.4	Ex
Coleoptera	Dryophthoridae	*Sphenophorus abbreviatus* (Fabricius)	Ex
Coleoptera	Elateridae	*Aeolus melliculus* Tarnier	Ex
Coleoptera	Elateridae	*Heteroderes vagus* Candèze	Ex
Coleoptera	Elateridae	*Melanotus dichrous* Erichson	Ex
Coleoptera	Endomychidae	*Holoparamecus caularum* (Aubé)	Ex
Coleoptera	Hydrophilidae	*Cercyon* sp.1	Ex
Coleoptera	Hydrophilidae	*Sphaeridium bipustulatum* (Fabricius)	Ex
Coleoptera	Hydrophilidae	*Cercyon haemorrhoidalis* (Fabricius)	Ex
Coleoptera	Laemophloeidae	Gen. sp.3	Ex
Coleoptera	Monotomidae	*Monotoma* sp.1	Ex
Coleoptera	Mycetophagidae	*Typhaea stercorea* (Linnaeus)	Ex
Coleoptera	Nitidulidae	*Stelidota geminata* (Say)	Ex
Coleoptera	Nitidulidae	*Epuraea biguttata* (Thunberg)	Ex
Coleoptera	Nitidulidae	*Carpophilus fumatus* (Boheman)	Ex
Coleoptera	Nitidulidae	*Carpophilus* sp.1	Ex
Coleoptera	Nitidulidae	*Phenolia limbata* (Boheman)	Ex
Coleoptera	Nitidulidae	*Carpophilus hemipterus* (Linnaeus)	Ex
Coleoptera	Nitidulidae	*Brachypeplus mauli* Gardner & Classey	Ex
Coleoptera	Nitidulidae	*Omosita colon* (Linnaeus)	Ex
Coleoptera	Nitidulidae	*Meligethes aeneus* (Fabricius)	Ex

Coleoptera	Nitidulidae	*Carpophilus* sp.3	Ex
Coleoptera	Phalacridae	Gen. sp.1	Ex
Coleoptera	Ptiliidae	*Ptenidium pusillum* (Gyllenhal)	Ex
Coleoptera	Ptiliidae	Gen. sp.1	Ex
Coleoptera	Rhizophagidae	Gen. sp.1	Ex
Coleoptera	Scarabaeidae	*Calamosternus granarius* (Linnaeus)	Ex
Coleoptera	Scarabaeidae	*Onthophagus taurus* (Schreber)	Ex
Coleoptera	Scarabaeidae	*Popillia japonica* Newman	Ex
Coleoptera	Scarabaeidae	*Onthophagus vacca* (Linnaeus)	Ex
Coleoptera	Silvanidae	*Cryptamorpha desjardinsii* (Guérin-Méneville)	Ex
Coleoptera	Staphylinidae	*Anotylus nitidifrons* (Wollaston)	Ex
Coleoptera	Staphylinidae	*Tachyporus chrysomelinus* (Linnaeus)	Ex
Coleoptera	Staphylinidae	*Atheta fungi* (Gravenhorst)	Ex
Coleoptera	Staphylinidae	*Tachyporus nitidulus* (Fabricius)	Ex
Coleoptera	Staphylinidae	*Cordalia obscura* (Gravenhorst)	Ex
Coleoptera	Staphylinidae	*Amischa analis* (Gravenhorst)	Ex
Coleoptera	Staphylinidae	*Aleochara bipustulata* (Linnaeus)	Ex
Coleoptera	Staphylinidae	*Oligota parva* Kraatz	Ex
Coleoptera	Staphylinidae	*Atheta aeneicollis* (Sharp)	Ex
Coleoptera	Staphylinidae	*Gyrohypnus fracticornis* (Muller)	Ex
Coleoptera	Staphylinidae	*Trichiusa immigrata* Lohse	Ex
Coleoptera	Staphylinidae	*Xantholinus longiventris* Heer	Ex
Coleoptera	Staphylinidae	*Anotylus nitidulus (Gravenhorst)*	Ex
Coleoptera	Staphylinidae	*Phloeonomus punctipennis* Thomson	Ex
Coleoptera	Staphylinidae	*Paraphloeostiba gayndahensis* (MacLeay)	Ex
Coleoptera	Staphylinidae	*Atheta amicula* (Stephens)	Ex
Coleoptera	Staphylinidae	*Anotylus* sp.2	Ex
Coleoptera	Staphylinidae	*Gabrius nigritulus* (Gravenhorst)	Ex
Coleoptera	Staphylinidae	*Philonthus politus* (Linnaeus)	Ex
Coleoptera	Staphylinidae	*Coproporus pulchellus* (Erichson)	Ex
Coleoptera	Staphylinidae	*Euplectus infirmus* (Raffray)	Ex
Coleoptera	Staphylinidae	Gen. sp.13	Ex
Coleoptera	Throscidae	*Trixagus* sp.2	Ex
Coleoptera	Trogidae	*Trox scaber* (Linnaeus)	Ex
Coleoptera	Carabidae	*Olisthopus inclavatus* Istaelson	Ind*
Coleoptera	Ciidae	*Atlantocis gillerforsi* Israelson	Ind*
Coleoptera	Curculionidae	*Pseudechinosoma nodosum* Hustache	Ind*
Coleoptera	Curculionidae	*Drouetius borgesi* Machado	Ind*
Coleoptera	Curculionidae	*Caulotrupis parvus* Israelson	Ind*
Coleoptera	Curculionidae	*Donus multifidus* (Israelson)	Ind*
Coleoptera	Curculionidae	*Drouetius azoricus* (Machado, 2009)	Ind*
Coleoptera	Dytiscidae	*Hydroporus guernei* Régimbart	Ind*
Coleoptera	Elateridae	*Heteroderes azoricus* (Tarnier)	Ind*

Coleoptera	Elateridae	*Alestrus dolosus* (Crotch)	Ind*
Coleoptera	Elateridae	*Athous pomboi* Platia & Borges	Ind*
Coleoptera	Elateridae	*Athous azoricus* Platia & Gudenzi	Ind*
Coleoptera	Lathridiidae	*Metophthalmus occidentalis* Israelson	Ind*
Coleoptera	Leiodidae	*Catops velhocabrali* Blas & Borges	Ind*
Coleoptera	Zopheridae	*Tarphius pomboi* Borges	Ind*
Coleoptera	Zopheridae	*Tarphius wollastoni* Crotch	Ind*
Coleoptera	Zopheridae	*Tarphius serranoi* Borges	Ind*
Coleoptera	Zopheridae	*Tarphius depressus* Gillerfors	Ind*
Coleoptera	Zopheridae	*Tarphius rufonodulosus* Israelson	Ind*
Coleoptera	Anthicidae	*Hirticollis quadriguttatus* (Rossi)	Ind
Coleoptera	Brentidae	*Aspidapion radiolus* (Wollaston)	Ind
Coleoptera	Carabidae	*Ocys harpaloides* (Audinet-Serville)	Ind
Coleoptera	Carabidae	*Calosoma olivieri* Dejean	Ind
Coleoptera	Carabidae	*Stenolophus teutonus* (Schrank)	Ind
Coleoptera	Carabidae	*Pterostichus aterrimus* (Herbst)	Ind
Coleoptera	Carabidae	*Acupalpus dubius* Schilsky	Ind
Coleoptera	Carabidae	*Microlestes negrita* (Wollaston)	Ind
Coleoptera	Carabidae	*Notiophilus quadripunctatus* Dejean	Ind
Coleoptera	Chrysomelidae	*Psylliodes marcidus* (Illiger)	Ind
Coleoptera	Chrysomelidae	*Chrysolina bankii* (Fabricius)	Ind
Coleoptera	Coccinellidae	*Rhyzobius litura* (Fabricius)	Ind
Coleoptera	Coccinellidae	*Scymnus interruptus* (Goeze) and *Scymnus nubilus* Mulsant	Ind
Coleoptera	Corylophidae	Gen. sp.1	Ind
Coleoptera	Curculionidae	*Pseudophloeophagus tenax* (Wollaston)	Ind
Coleoptera	Curculionidae	*Cathormiocerus curvipes* (Wollaston)	Ind
Coleoptera	Curculionidae	*Orthochaetes insignis* (Aubé)	Ind
Coleoptera	Curculionidae	*Pseudophloeophagus aenopiceus* (Boheman)	Ind
Coleoptera	Dasytidae	*Psilothrix viridicoerulea* (Geoffroy)	Ind
Coleoptera	Dryopidae	*Dryops luridus* (Erichson)	Ind
Coleoptera	Dryopidae	*Dryops algiricus* Lucas	Ind
Coleoptera	Dytiscidae	*Agabus bipustulatus* (Linnaeus)	Ind
Coleoptera	Laemophloeidae	*Placonotus* sp.1	Ind
Coleoptera	Laemophloeidae	*Cryptolestes* sp.1	Ind
Coleoptera	Leiodidae	*Catops coracinus* Kellner	Ind
Coleoptera	Phalacridae	*Stilbus testaceus* (Panzer)	Ind
Coleoptera	Scraptiidae	*Anaspis proteus* (Wollaston)	Ind
Coleoptera	Scydmaenidae	*Cephennium distinctum* Besuchet	Ind
Coleoptera	Staphylinidae	*Rugilus orbiculatus* (Paykull)	Ind
Coleoptera	Staphylinidae	*Quedius curtipennis* Bernhauer	Ind
Coleoptera	Staphylinidae	*Ocypus olens* (Muller)	Ind

Coleoptera	Staphylinidae	*Ocypus aethiops* (Waltl)	Ind
Coleoptera	Staphylinidae	*Carpelimus corticinus* (Gravenhorst)	Ind
Coleoptera	Staphylinidae	*Proteinus atomarius* Erichson	Ind
Coleoptera	Staphylinidae	*Quedius simplicifrons* (Fairmaire)	Ind
Coleoptera	Staphylinidae	*Astenus lyonessius* (Joy)	Ind
Coleoptera	Staphylinidae	*Phloeonomus* sp.1	Ind
Coleoptera	Staphylinidae	*Pseudoplectus perplexus* (Jacquelin du Val)	Ind
Coleoptera	Staphylinidae	*Phloeonomus* sp. 4	Ind
Coleoptera	Staphylinidae	*Sepedophilus lusitanicus* (Hammond)	Ind
Coleoptera	Staphylinidae	*Scopaeus portai* Luze	Ind
Coleoptera	Staphylinidae	*Tachyporus* sp.1	Ind
Dermaptera	Anisolabididae	*Euborellia annulipes* (Lucas)	Ex
Dermaptera	Forficulidae	*Forficula auricularia* Linnaeus	Ex
Geophilomorpha	Geophilidae	*Geophilus truncorum* Bergsoe & Meinert	Ind
Hemiptera	Anthocoridae	*Buchananiella continua* (White)	Ex
Hemiptera	Aphididae	*Rhopalosiphonimus latysiphon* (Davidson)	Ex
Hemiptera	Aphididae	*Rhopalosiphum oxyacanthae* (Schrank)	Ex
Hemiptera	Aphididae	*Neomyzus circumflexus* (Buckton)	Ex
Hemiptera	Aphididae	*Dysaphis plantaginea* (Passerini)	Ex
Hemiptera	Aphididae	*Toxoptera aurantii* (Boyer de Fonscolombe)	Ex
Hemiptera	Coccidae	Gen. sp.4	Ex
Hemiptera	Coccidae	Gen. sp.1	Ex
Hemiptera	Coccidae	Gen. sp.2	Ex
Hemiptera	Coccidae	Gen. sp.3	Ex
Hemiptera	Psyllidae	Gen. sp.2	Ex
Hemiptera	Reduviidae	*Triatoma rubrofasciata* (De Geer)	Ex
Hemiptera	Cicadellidae	*Aphrodes hamiltoni* Quartau & Borges	Ind*
Hemiptera	Cixiidae	*Cixius azoterceirae* Remane & Asche	Ind*
Hemiptera	Cixiidae	*Cixius azopifajo* Remane & Asche	Ind*
Hemiptera	Cixiidae	*Cixius azofloresi* Remane & Asche	Ind*
Hemiptera	Cixiidae	*Cixius azomariae* Remane & Asche	Ind*
Hemiptera	Lygaeidae	*Nysius atlantidum* Horváth	Ind*
Hemiptera	Aleyrodidae	Gen. sp.1	Ind
Hemiptera	Anthocoridae	*Brachysteles parvicornis* (A. Costa)	Ind
Hemiptera	Aphididae	*Acyrthosiphon pisum* Harris	Ind
Hemiptera	Aphididae	*Pseudacaudella rubida* (Börner)	Ind
Hemiptera	Cicadellidae	*Anoscopus albifrons* (Linnaeus)	Ind
Hemiptera	Cicadellidae	*Euscelidius variegatus*	Ind

		(Kirschbaum)	
Hemiptera	Cicadellidae	Gen. sp.1	Ind
Hemiptera	Cicadellidae	Gen. sp.4	Ind
Hemiptera	Cydnidae	*Geotomus punctulatus* (Costa)	Ind
Hemiptera	Delphacidae	*Megamelodes quadrimaculatus* (Signoret)	Ind
Hemiptera	Delphacidae	*Kelisia ribauti* Wagner	Ind
Hemiptera	Delphacidae	Gen. sp.4	Ind
Hemiptera	Flatidae	*Cyphopterum adcendens* (Herr.-Schaff.)	Ind
Hemiptera	Lachnidae	*Cinara juniperi* (De Geer)	Ind
Hemiptera	Lygaeidae	*Scolopostethus decoratus* (Hahn)	Ind
Hemiptera	Lygaeidae	*Microplax plagiata* (Fieber)	Ind
Hemiptera	Lygaeidae	*Plinthisus brevipennis* (Latreille)	Ind
Hemiptera	Lygaeidae	*Kleidocerys ericae* (Horváth)	Ind
Hemiptera	Lygaeidae	*Beosus maritimus* (Scopoli)	Ind
Hemiptera	Lygaeidae	*Eremocoris maderensis* (Wollaston)	Ind
Hemiptera	Lygaeidae	*Aphanus rolandri* (Linnaeus)	Ind
Hemiptera	Lygaeidae	*Emblethis denticollis* Horváth	Ind
Hemiptera	Microphysidae	*Loricula coleoptrata* (Fallén)	Ind
Hemiptera	Miridae	*Pithanus maerkelii* (Herrich-Schaeffer)	Ind
Hemiptera	Nabidae	*Nabis pseudoferus* Remane	Ind
Hemiptera	Tingidae	*Acalypta parvula* (Fallén)	Ind
Hemiptera	Triozidae	*Trioza laurisilvae* Hodkinson	Ind
Julida	Blaniulidae	*Blaniulus guttullatus* (Fabricius)	Ex
Julida	Blaniulidae	Choneiulus palmatus (Nemec)	Ex
Julida	Blaniulidae	*Proteroiulus fuscus* (Am Stein)	Ex
Julida	Blaniulidae	*Nopoiulus kochii* (Gervais)	Ex
Julida	Julidae	*Ommatoiulus moreletii* (Lucas)	Ex
Julida	Julidae	*Cylindroiulus propinquus* (Porat)	Ex
Julida	Julidae	*Brachyiulus pusillus* (Leach)	Ex
Julida	Julidae	*Cylindroiulus latestriatus* (Curtis)	Ex
Lithobiomorpha	Lithobiidae	*Lithobius pilicornis* Newport	Ind
Lithobiomorpha	Lithobiidae	*Lithobius* sp.2	Ind
Microcoryphia	Machilidae	*Trigoniophthalmus borgesi* Mendes et al.	Ind*
Microcoryphia	Machilidae	*Dilta saxicola* (Womersley)	Ind
Neuroptera	Hemerobiidae	Gen. sp.1	Ind
Opiliones	Phalangiidae	*Leiobunum blackwalli* Meade	Ind
Opiliones	Phalangiidae	*Homalenotus coriaceus* (Simon)	Ind
Orthoptera	Gryllidae	*Gryllus bimaculatus* (De Geer)	Ex
Orthoptera	Gryllidae	*Eumodicogryllus bordigalensis* (Latreille)	Ex
Orthoptera	Gryllidae	Gen. sp.1	Ex
Polydesmida	Paradoxosomatidae	*Oxidus gracilis* (C.L.Koch)	Ex
Polydesmida	Polydesmidae	*Polydesmus coriaceus* Porat	Ex
Pseudoscorpiones	Chthoniidae	*Chthonius ischnocheles* (Hermann)	Ex

Pseudoscorpiones	Chthoniidae	*Chthonius tetrachelatus* (Preyssler)	Ex
Pseudoscorpiones	Neobisiidae	*Neobisium maroccanum* Beier	Ind
Psocoptera	Ectopsocidae	*Ectopsocus briggsi* McLachlan	Ex
Psocoptera	Lachesillidae	*Lachesilla greeni* (Pearman)	Ex
Psocoptera	Elipsocidae	*Elipsocus azoricus* Meinander	Ind*
Psocoptera	Peripsocidae	*Peripsocus subfasciatus* (Rambur)	Ind
Scolopendromorpha	Cryptopidae	*Cryptops hortensis* Leach	Ind
Scutigeromorpha	Scutigeridae	*Scutigera coleoptrata* (Linnaeus)	Ex
Symphyla	Scutigerellidae	*Scutigerella immaculata* (Newport)	Ind
Thysanoptera	Phlaeothripidae	*Nesothrips propinquus* (Bagnall)	Ex
Thysanoptera	Phlaeothripidae	*Hoplothrips corticis* (De Geer)	Ind
Thysanoptera	Thripidae	*Ceratothrips ericae* (Haliday)	Ind

References:

1. Cardoso P, Rigal F, Fattorini S, Terzopoulou S, Borges PAV (2013) Integrating landscape disturbance and indicator species in conservation studies. PLoS One 8: e63294.

2. Borges PAV, Aguiar C, Amaral J, Amorin IR, André G, et al. (2005) Ranking protected areas in the Azores using standardized sampling of soil epigean arthropods. Biodivers Conserv 14: 2029–2060.

3. Meijer SS, Whittaker RJ, Borges PAV (2011) The effects of land-use change on arthropod richness and abundance on Santa Maria Island (Azores): unmanaged plantations favour endemic beetles. J Insect Conserv 15: 505–522.

4. Cardoso P, Aranda SC, Lobo JM, Dinis F, Gaspar C, Borges PAV (2009) A spatial scale assessment of habitat effects on arthropod communities of an oceanic island. Acta Oecologica 35: 590–597.

5. Oliver I, Beattie AJ (1996) Invertebrate morphospecies as surrogates for species: a case study. Conserv Biol 10(1): 99–109.

6. R Development Core Team 2012. R: A language and environment for statistical computing. – R Foundation for Statistical Computing, Vienna, Austria. ISBN 3-

900051-07-0, URL http://www.R-project.org

7. Oksanen J, Blanchet FG, Kindt R, Legendre P, Minchin PR et al. (2013) vegan: Community Ecology Package. R package version 2.0-10. http://CRAN.R-project.org/package=vegan
