# Supplementary material for: The Colonisation of Exotic Species Does Not Have to Trigger Faunal Homogenisation: Lessons from the Assembly Patterns of Arthropods on Oceanic Islands
Source: PLoS One. 2015 May 29;10(5):e0128276. doi: 10.1371/journal.pone.0128276 (PMC4449220; doi:10.1371/journal.pone.0128276)
Supplement: S2 File — (PDF) [file pone.0128276.s002.pdf]

**S2 File. Description of the five null models used to determine the significance of nested/anti-nested patterns on raw data and rarefied matrices; we selected from least to most restrictive null models in detecting nested patterns.**

To properly detect nested/anti-nested patterns and better understand the underlying mechanisms, we used a combination of five different null models as follows: i) the equiprobable–equiprobable (EE) null model, assuming equal probabilities of occurrences among matrix cells and controlled only for the total of occurrences. This is the less restrictive null model that incurs more Type I errors, i.e., showing a high proportion of non-nested matrices to be nested [1-2]; ii) the fixed row and equiprobable column totals (FE) null model, which preserves the total species occurrences (i.e., species incidences) but allows species richness to vary randomly and equiprobably per site [3]. This null model was thus used to assess the contribution of species to the observed pattern (i.e. $NODF_{species}$); iii) the equiprobable row and fixed column totals (EF) preserve the species richness (i.e., species composition) per site but allow species occurrences to vary randomly and equiprobably [3]. This null model was thus used to assess the contribution of transects to the observed pattern (i.e., $NODF_{transects}$); iv) the proportional–proportional (PP) null model, in which presences per row and column vary randomly, but the mean row and column totals are unbiased and match those of the original matrix. This null model is considered the most realistic and mimics random colonisations in a metacommunity of small-scale surveys in which occurrences are expected to vary substantially [2]. In addition, it is considered a restrictive null model, with sufficient power to discriminate nested from segregated patterns; iii) v) a null model (RC) based on abundance data, to control the passive sampling effect on the presence–absence data. The widespread distribution of abundant species in contrast to

the narrow distribution of rare species can increase the degree of nestedness through a passive sampling effect [1], [4-5]. The RC null model randomly adds species between transects, reducing the effect of the widespread abundant species to the detriment of rare species. In the RC null model, individuals are assigned to matrix cells in proportion to observed row and column abundance totals until total abundances are reached for each row and column [6]. Because abundance-based rarefaction randomises individuals within transects and the RC null model does the same between transects, we did not perform the RC null model on the rarefied matrices.

**References**

1. Ulrich, W, Almeida-Neto M, Gotelli NJ (2009) A consumer's guide to nestedness analysis. Oikos 118: 3-17.

2. Ulrich W, Gotelli NJ (2012) A null model algorithm for presence–absence matrices based on proportional resampling. Ecol Model 244: 20-27.

3. Ulrich W, Gotelli NJ (2007) Null model analysis of species nestedness patterns. Ecology 88: 1924-1831.

4. Wright DH, Patterson BD, Mikkelson GM, Cutler A, Atmar W (1998) A comparative analysis of nested subset patterns of species composition. Oecologia 113: 1-20.

5. McAbendroth L, Foggo A, Rundle SD, Bilton DT (2005) Unravelling nestedness and spatial pattern in pond assemblages. J Anim Ecol 74: 41-49.

6. Ulrich W, Gotelli NJ (2010) Null model analysis of species associations using abundance data. Ecology 91: 3384-3397.
