# Supplementary material for: The Colonisation of Exotic Species Does Not Have to Trigger Faunal Homogenisation: Lessons from the Assembly Patterns of Arthropods on Oceanic Islands
Source: PLoS One. 2015 May 29;10(5):e0128276. doi: 10.1371/journal.pone.0128276 (PMC4449220; doi:10.1371/journal.pone.0128276)
Supplement: S3 File — An index of species replacement was also calculated to compare the β-diversity of whole assemblages (including indigenous and exotic species) and of the indigenous species alone (Table H). (PDF) [file pone.0128276.s003.pdf]

S3 File. Degree of nestedness (NODF) calculated on the raw data and on the ten rarefied matrices (1–10) using presence–absence data of the whole assemblages, and of both indigenous and exotic species separately, for the considered regional data (Table B and Table C) and also separately for the four study islands (Table D and Table E on rarefied and raw data, respectively) and the four study habitats (Table F and Table G, on rarefied and raw data, respectively). An index of species replacement was also calculated to compare the β-diversity of whole assemblages (including indigenous and exotic species) and of the indigenous species alone (Table H).

Table B. Degree of nestedness calculated on presence–absence data using the raw data ($NODF_{raw}$) and the ten rarefied matrices ($NODF_{rarefied}$, the mean degree of nestedness and SD are indicated), for the whole assemblages and considering indigenous and exotic species separately. The $NODF_{transect}$ and $NODF_{species}$ values indicate the contribution of transects and species, respectively, to the observed pattern. The 95% confidence interval of five null models after 1,000 permutations are indicated (EE is the equiprobable–equiprobable null model, PP is the proportional–proportional null model, FE is the fixed row and equiprobable column totals, EF is the equiprobable row and fixed column totals, RC is the null model controlling the passive sampling effect, see Methods for more details). The P values are indicated for the raw data; P < 0.001 indicates significant nestedness, P > 0.999 indicates significant anti-nestedness. The number of rarefied matrices that were significant at P < 0.05 for nestedness, and P > 0.95 for anti-nestedness, is also indicated (see Table C in S3 File, for detailed values).

**Data**		**Null**			**Means**		**Null**		
**Metric**	**$NODF_{raw}$**	**models**	**95% IC**	**P**	**$NODF_{rarefied}$**	**SD**	**models**	**P < 0.05**	**P > 0.95**
**Whole assemblages**									
NODF	18.50	EE	10.60–11.23	<0.001	13.62	0.41	EE	10	0
		PP	18.15–19.79	0.172			PP	0	1
		RC	48.03–52.21	>0.999					
$NODF_{transects}$	34.79	EF	11.65–12.25	<0.001	24.57	0.51	EF	10	0
		PP	33.07–37.54	0.322			PP	0	0
		RC	75.77–78.82	>0.999					

$NODF_{species}$	17.71	FE	15.34–15.82	<0.001	12.18	0.39	FE	7	0
		PP	17.38–19.00	0.157			PP	0	1
		RC	46.71–50.94	>0.999					
**Indigenous species**									
NODF	16.25	EE	8.83–8.84	<0.001	14.06	1.11	EE	10	0
		PP	15.47–17.73	0.302			PP	0	0
		RC	48.35–53.29	>0.999					
$NODF_{transects}$	26.62	EF	9.00–9.85	<0.001	19.66	1.48	EF	10	0
		PP	23.93–31.37	0.35			PP	0	0
		RC	72.29–76.67	>0.999					
$NODF_{species}$	13.72	FE	12.53–13.54	<0.01	10.42	0.85	FE	4	0
		PP	13.25–14.64	0.272			PP	0	0
		RC	42.26–47.75	>0.999					
**Exotic species**									
NODF	25.50	EE	11.68–12.62	<0.001	19.78	0.63	EE	10	0
		PP	24.01–27.35	0.458			PP	0	0
		RC	53.13–58.61	>0.999					
$NODF_{transects}$	43.7	EF	13.81–14.71	<0.001	30.70	0.93	EF	10	0
		PP	39.66–46.63	0.308			PP	0	0
		RC	76.37–80.53	>0.999					

$NODF_{species}$	22.62	FE	17.17–18.04	<0.001	14.97	0.49	FE	10	0
		PP	21.41–24.43	0.486			PP	0	0
		RC	49.35–55.13	>0.999					

Table C.- Degree of nestedness calculated in the ten rarefied matrices (ranged 1-10), using the presence-absence data for the whole assemblages, and for both indigenous and exotic species separately. $NODF_{transect}$ and $NODF_{species}$ values indicate the contribution of transects and species, respectively, to the observed pattern. Intervals of confidence at 95% of five null models after 1000 permutations are indicated (EE is the equiprobable-equiprobable null model, PP is the proportional-proportional null model, FE is the fixed raw and equiprobable column totals, EF is the equiprobable raw and fixed column totals, RC is the null model controlling the passive sampling effect, see Methods for more details). All significant P indicated nestedness (P <0.001; P <0.01; P <0.05), except the first rarefied matrix of the whole assemblages using the PP null model (P >0.95), which indicated anti-nestedness.

	**Null models**	**$NODF_{rarefied}$**	**P**	**$NODF_{rarefied}$**	**P**	**$NODF_{rarefied}$**	**P**	**$NODF_{rarefied}$**	**P**	**$NODF_{rarefied}$**	**P**
**Whole assemblages**		1		2		3		4		5	
NODF		12.73		13.65		13.71		14.03		13.34	
	EE 95% IC	7.78-8.56	<0.001	7.93-8.70	<0.001	8.32-9.11	<0.001	8.23-9.04	<0.001	7.87-8.70	<0.001
	PP 95% IC	12.69-13.81	>0.95	13.22-14.53	0.269	13.42-14.70	0.166	13.62-15.04	0.216	13.11-14.30	0.14
$NODF_{transects}$		23.74		24.97		24.48		24.62		24.68	
	EF 95% IC	7.66-8.34	<0.001	7.94-8.64	<0.001	8.17-8.87	<0.001	6.75-8.07	<0.001	8.03-8.73	<0.001
	PP 95% IC	22.32-26.47	0.278	23.21-27.75	0.341	22.63-27.02	0.421	22.71-27.62	0.377	22.96-27.38	0.388
$NODF_{species}$		11.36		12.22		12.19		12.55		11.91	
	FE 95% IC	11.00-11.76	0.447	11.27-11.98	<0.01	11.61-12.36	0.107	11.57-12.35	<0.01	11.10-11.81	<0.01
	PP 95% IC	11.42-12.32	>0.95	11.87-12.96	0.288	12.04-13.05	0.101	12.25-13.42	0.188	11.78-12.75	0.08
**Indigenous species**											
NODF		11.74		14.43		14.16		13.98		13.62	

	EE 95% IC	5.92-7.15	<0.001	6.74-8.09	<0.001	6.89-8.32	<0.001	6.37-7.65	<0.001	6.60-7.89	<0.001
	PP 95% IC	11.14-14.23	0.126	12.84-16.26	0.48	13.19-17.29	0.204	12.49-16.64	0.351	12.72-16.28	0.208
$NODF_{transects}$		17.62		19.81		19.39		19.51		17.96	
	EF 95% IC	5.84-6.83	<0.001	7.03-8.14	<0.001	7.03-8.33	<0.001	6.68-7.88	<0.001	6.93-8.20	<0.001
	PP 95% IC	15.72-22.64	0.229	16.55-23.90	0.46	17.20-25.55	0.247	16.52-24.80	0.311	16.25-23.50	0.354
$NODF_{species}$		8.5		10.91		10.35		10.26		10.77	
	FE 95% IC	8.05-9.49	0.244	17.06-20.52	0.11	9.43-11.04	0.338	16.69-20.32	0.116	8.96-10.56	<0.01
	PP 95% IC	13.33-15.23	0.388	10.09-11.67	0.462	9.98-11.68	0.156	9.64-11.43	0.376	10.12-11.98	0.192
**Exotic species**											
NODF		20.38		19.26		19.83		20.66		18.93	
	EE 95% IC	9.12-10.30	<0.001	8.50-9.70	<0.001	9.01-10.25	<0.001	9.67-10.84	<0.001	8.53-9.69	<0.001
	PP 95% IC	18.25-21.51	0.296	17.34-20.55	0.327	18.07-21.51	0.422	18.70-22.62	0.461	17.38-20.52	0.431
$NODF_{transects}$		31.37		31		30.24		31.36		30.58	
	EF 95% IC	9.78-10.87	<0.001	8.93-9.98	<0.001	9.63-10.72	<0.001	10.27-11.31	<0.001	8.99-9.99	<0.001
	PP 95% IC	26.80-34.42	0.334	26.83- 34.67	0.388	26.22- 34.24	0.439	26.23-35.05	0.292	26.56-33.97	0.381
$NODF_{species}$		15.42		14.49		15.03		15.5		14.19	
	FE 95% IC	12.93-14.27	<0.001	12.24-13.55	<0.001	12.69-14.05	<0.001	13.33-14.75	<0.001	12.17-13.49	<0.001
	PP 95% IC	14.16-16.10	0.246	13.33-15.23	0.256	14.03-15.97	0.407	14.86-17.10	0.254	13.39-15.29	0.442
**Whole assemblages**		6		7		8		9		10	
NODF		14		13.52		13.69		14.15		13.42	
	EE 95% IC	8.44-9.28	<0.001	8.10-8.92	<0.001	8.13-8.89	<0.001	8.38-9.21	<0.001	7.94-8.78	<0.001
	PP 95% IC	13.76-14.96	0.144	13.27-14.48	0.124	13.33- 14.62	0.221	13.86-15.22	0.14	13.10-14.21	0.217
$NODF_{transects}$		23.58		24.92		24.78		24.95		25	
	EF 95% IC	8.25-8.93	<0.001	7.95-8.63	<0.001	8.05-8.77	<0.001	8.28-8.98	<0.001	7.91-8.63	<0.001
	PP 95% IC	22.78-26.99	0.142	23.09-27.51	0.365	23.04-27.33	0.383	23.52-28.09	0.235	23.26-27.35	0.391

$NODF_{species}$		12.64		12.01		12.3		12.65		11.99	
	FE 95% IC	11.93-12.70	<0.05	11.50-12.25	0.195	11.47-12.19	<0.01	11.90-12.63	<0.05	11.25-11.98	<0.05
	PP 95% IC	12.36-13.39	0.2	11.89-12.86	0.081	13.01-13.12	0.199	12.41-13.55	0.154	11.79-12.69	0.181
**Indigenous species**											
NODF		15.45		15.08		13.1		15.24		13.82	
	EE 95% IC	7.07-8.56	<0.001	6.58-7.98	<0.001	6.63- 7.93	<0.001	6.67-8.10	<0.001	6.38-7.67	<0.001
	PP 95% IC	14.04-18.32	0.277	13.59-17.99	0.323	12.46-15.80	0.13	13.49-17.77	0.428	12.61-15.93	0.321
$NODF_{transects}$		20.24		22.16		18.63		21.87		19.45	
	EF 95% IC	7.43-8.71	<0.001	7.05-8.31	<0.001	6.73-7.89	<0.001	6.64-8.16	<0.001	6.86-8.13	<0.001
	PP 95% IC	17.80-26.13	0.263	18.51-28.26	0.414	16-79-24.20	0.194	18.16-26.67	0.456	17.02-24.26	0.291
$NODF_{species}$		11.77		10.68		9.75		10.68		10.49	
	FE 95% IC	9.74-11.51	<0.01	9.18-10.78	<0.05	9.23-10.72	0.351	9.33-11.05	0.114	8.78-10.34	<0.05
	PP 95% IC	10.88-12.94	0.444	10.27-12.06	0.178	9.61- 11.16	0.059	9.97-11.73	0.362	9.72-11.31	0.497
**Exotic species**											
NODF		19.13		19.63		20.18		20.55		19.27	
	EE 95% IC	9.08-10.34	<0.001	8.97-10.27	<0.001	8.94-10.16	<0.001	9.33-10.58	<0.001	8.85-10.0	<0.001
	PP 95% IC	17.68-20.85	0.454	17.98-21.33	0.472	18.32-21.44	0.345	18.78-22.24	0.438	17.92-20.84	0.45
$NODF_{transects}$		28.62		30.12		31.98		31.17		30.59	
	EF 95% IC	9.54-10.54	<0.001	9.25-10.33	<0.001	9.60-10.69	<0.001	9.98-11.02	<0.001	9.24-10.28	<0.001
	PP 95% IC	25.53- 32.69	0.417	26.44-33.90	0.465	27.67-34.91	0.305	27.07-34.87	0.437	27.45-34.31	0.458
$NODF_{species}$		14.94		14.8		15.17		15.66		14.46	
	FE 95% IC	12.96-14.31	<0.001	12.84- 14.24	<0.01	12.75-14.09	<0.001	13.30-14.65	<0.001	12.64-14.07	<0.01
	PP 95% IC	13.95-16.01	0.463	13.80-15.84	0.468	14.11-16.15	0.426	14.65-16.72	0.49	13.66-15.45	0.44

Table D.- Degree of nestedness (NODF) calculated separately on the four study islands for indigenous and for exotic species, using presence-absence data of the ten rarefied matrices (ranged 1-10). Intervals of confidence at 95% of the PP null model (proportional-proportional null model) after 1000 permutations are indicated. All the significant NODF values indicate a smaller degree of nestedness of the respective rarefied matrices than those obtained in all the 1000 null model permutations (P >0.999; P >0.99; P >0.95), indicating anti-nestedness.

	1 **NODF**	**95% IC**	**P**	2 **NODF**	**95% IC**	**P**	3 **NODF**	**95% IC**	**P**	4 **NODF**	**95% IC**	**P**	5 **NODF**	**95% IC**	**P**
**Indigenous species**															
**Flores**	25.76	22.57-29.50	0.497	23.43	20.86-27.68	0.332	28.57	24.78-32.66	0.481	24.74	21.71-29.78	0.375	29.01	25.53-34.51	0.371
**Terceira**	17.18	16.72-21.84	0.058	19.21	17.42-21.59	0.404	19.21	17.31-21.54	0.406	21.79	19.08-25.66	0.413	18.64	17.17-23.41	0.203
**Santa Maria**	14.65	14.42-17.92	>0.95	21.33	18.92-23.71	0.484	17.35	15.42-20.43	0.354	15.99	14.95-20.22	0.171	17.95	16.64-20.99	0.281
**Faial**	18.03	19.86-25.53	>0.999	21.56	21.31-27.58	>0.95	18.24	19.81-25.83	>0.999	24.20	23.36-31.86	0.085	20.21	20.39-26.37	>0.95
**Exotics species**															
**Flores**	30.88	28.88-36.35	0.237	33.22	30.14-36.31	0.454	29.19	26.75-34.61	0.348	30.23	28.67-36.82	0.169	32.11	29.30-36.92	0.365
**Terceira**	18.86	17.53-21.86	0.305	18.39	17.34-21.16	0.229	17.78	16.01-20.01	0.486	19.43	17.44-22.39	0.419	19.10	17.29-21.41	0.464
**Santa Maria**	24.68	25.81-29.98	>0.999	22.89	23.70-28.95	>0.99	25.68	25.56-29.81	>0.99	24.44	24.88-29.51	>0.99	23.79	24.43-28.96	>0.99
**Faial**	26.04	23.79-29.21	0.48	26.66	24.48-29.75	0.433	27.08	24.47-30.34	0.446	29.20	26.22-33.32	0.446	24.22	23.18-28.35	0.165
	6			7			8			9			10		
**Indigenous species**															
**Flores**	27.08	23.34-31.51	0.468	23.43	20.86-27.68	0.332	27.23	23.72-30.88	0.419	29.75	25.80-33.72	0.419	24.41	21.15-28.09	0.489
**Terceira**	20.06	19.74-25.48	>0.95	23.9	21.37-27.87	0.339	16.99	16.65-21.07	0.054	21.78	19.63-25.48	0.346	18.74	16.71-21.77	0.392
**Santa Maria**	21.24	19.77-25.32	0.231	18.72	17.56-23.21	0.162	16.12	15.98-19.82	>0.95	18.18	16.58-21.00	0.323	24.41	21.31-28.48	0.471
**Faial**	25.98	25.74-34.56	>0.95	20.25	20.73-27.45	>0.99	21.85	21.76-28.83	>0.95	19.55	20.75-26.26	>0.99	21.88	22.27-28.71	>0.95
**Exotics species**															
**Flores**	27.41	26.22-31.70	0.168	28.15	26.51-32.68	0.258	28.6	26.32-31.89	0.420	32.2	29.69-38.79	0.267	33.04	29.73-39.05	0.469
**Terceira**	18.88	17.66-22.13	0.209	17.66	16.60-20.52	0.231	19.25	18.28-22.56	0.173	20.3	18.80-23.76	0.291	19.07	17.51-20.96	0.486

**Santa Maria**	23.67	23.47-27.93	>0.95	23.65	23.99-28.08	>0.99	25.23	24.43-28.36	0.154	26.37	26.29-31.35	>0.95	25.08	25.89-30.57	>0.999
**Faial**	25.54	22.85-28.60	0.439	28.93	25.59-32.02	0.405	29.87	26.84-33.61	0.469	25.11	22.64-28.90	0.493	24.97	23.79-29.42	0.157

Table E. Degree of nestedness calculated for the four study islands using presence–absence matrices of the raw data ($NODF_{raw}$), for indigenous and exotic species, separately. The 95% confidence intervals of two null models after 1,000 permutations are indicated (PP is the proportional–proportional null model and RC is the null model controlling the passive sampling effect). All the significant NODF values indicate a lower degree of nestedness than those obtained in all the 1,000 null model permutations (P > 0.999; P > 0.95), indicating anti-nestedness.

**Species origin**				
**Island**	**$NODF_{raw}$**	**Null models**	**95% IC**	**P**
**Indigenous**				
**Flores**	29.60	PP	28.27–33.24	0.204
		RC	57.99–65.99	>0.999
**Terceira**	20.33	PP	19.89–23.24	0.084
		RC	63.77–75.29	>0.999
**Santa Maria**	26.72	PP	26.92–30.28	>0.999
		RC	64.09–73.63	>0.999
**Faial**	22.89	PP	22.76–27.05	>0.95
		RC	51.95–62.59	>0.999
**Exotics**				
**Flores**	42.28	PP	40.14–48.20	0.213
		RC	67.38–77.13	>0.999
**Terceira**	32.51	PP	29.34–36.72	0.472

		RC	62.75–73.87	>0.999
**Santa Maria**	16.24	PP	16.61–18.79	>0.999
		RC	29.16–32.01	>0.999
**Faial**	34.66	PP	31.94–39.36	0.397
		RC	59.44–70.57	>0.999

Table F.- Degree of nestedness (NODF) calculated on the four considered habitats for the whole assemblages and separately for indigenous and for exotic species, using presence-absence data of the ten rarefied matrices (ranged 1-10). Intervals of confidence at 95% of the PP null model (proportional-proportional null model) after 1000 permutations are indicated. All the significant NODF values indicate a smaller degree of nestedness of the respective rarefied matrices than those obtained in all the 1000 null model permutations (P >0.999; P >0.99; P >0.95), indicating anti-nestedness.

	1 NODF	95% IC	P	2 NODF	95% IC	P	3 NODF	95% IC	P	4 NODF	95% IC	P	5 NODF	95% IC	P
**Whole assemblages**															
**Native forest**	21.74	21.94-24.19	>0.99	23.38	23.17-25.79	0.062	22.68	22.96-25.66	>0.99	24.48	23.64-27.53	0.195	24.57	24.12-27.25	0.090
**Semi-natural pastures**	23.82	25.74-30.32	>0.999	23.09	22.84-26.06	0.056	22.01	21.83-25.04	>0.95	24.58	24.52-27.86	>0.95	23.55	22.60-26.57	0.190
**Exotic forest**	26.54	26.83-30.85	>0.99	29.63	29.30-33.91	0.054	31.41	30.35-35.34	0.193	29.95	29.10-33.79	0.109	29.14	29.21-33.52	>0.95
**Intensive pastures**	22.04	22.17-24.99	>0.95	23.24	22.34-27.54	0.162	22.33	21.93-24.95	0.118	23.44	23.55-27.07	>0.95	22.82	22.66-26.08	>0.95
**Indigenous species**															
**Native forest**	18.30	18.00-21.09	0.069	22.00	21.95-25.86	>0.95	21.35	21.50-25.72	>0.95	26.26	24.87-30.52	0.210	23.15	23.46-28.48	>0.95
**Semi-natural pastures**	27.29	26.09-33.02	0.148	24.48	21.60-28.31	0.464	23.26	21.69-27.14	0.207	24.14	22.50-29.66	0.185	26.34	23.59-31.84	0.261
**Exotic forest**	19.54	18.81-25.18	0.073	24.77	20.62-29.75	0.450	24.45	23.29-33.32	0.09	22.60	19.09-26.74	0.473	23.61	22.05-29.63	0.131
**Intensive pastures**	16.14	14.44-19.64	0.288	21.81	19.23-27.73	0.292	21.04	18.24-25.46	0.408	18.16	15.64-22.48	0.353	18.22	14.95-21.55	0.485
**Exotics species**															
**Native forest**	30.19	28.36-33.51	0.366	26.79	25.06-29.94	0.352	26.25	26.10-31.23	>0.95	26.20	24.31-29.49	0.321	28.06	25.79-31.03	0.497
**Semi-natural pastures**	24.38	25.96-32.91	>0.999	24.75	23.86-29.78	0.111	26.12	23.54-30.33	0.421	27.81	27.08-32.64	0.091	27.81	27.08-32.46	0.088
**Exotic forest**	30.19	28.20-33.46	0.325	40.94	39.34-48.87	0.122	42.17	39.97-49.06	0.199	40.75	38.92-47.24	0.171	36.48	37.69-46.54	>0.99
**Intensive**	25.32	24.70-28.94	0.100	23.90	23.18-28.30	0.121	24.48	23.46-27.36	0.260	26.41	26.54-31.62	>0.95	26.06	25.28-30.32	0.105

	6			7			8			9			10		
**pastures**															
**Whole assemblages**															
**Native forest**	24.07	23.57-26.35	0.140	23.32	22.83-25.83	0.106	25.57	25.26-28.73	0.075	23.03	23.09-25.96	>0.95	24.26	24.36-27.40	>0.95
**Semi-natural pastures**	23.50	23.26-27.06	0.052	24.46	23.82-27.27	0.143	26.16	25.90-29.75	0.060	24.83	23.96-27.40	0.175	24.09	23.08-26.68	0.239
**Exotic forest**	29.07	29.09-33.18	>0.95	28.26	27.92-31.70	0.065	27.07	27.05-31.48	>0.95	31.08	30.74-35.13	0.069	31.10	29.97-36.10	0.177
**Intensive pastures**	23.88	23.55-27.55	0.074	22.59	22.50-25.43	>0.95	25.63	24.93-29.13	0.146	25.47	24.96-29.01	0.086	21.99	21.98-24.99	>0.95
**Indigenous species**															
**Native forest**	25.60	24.88-29.64	0.089	24.80	24.12-28.52	0.117	23.26	23.15-27.71	>0.95	21.07	21.01-24.70	>0.95	23.63	23.32-27.15	0.052
**Semi-natural pastures**	22.71	22.21-29.56	0.056	25.83	23.05-30.85	0.412	24.38	22.96-28.77	0.184	32.71	27.87-37.18	0.407	24.80	23.42-30.64	0.133
**Exotic forest**	26.24	22.25-31.08	0.488	23.25	20.61-27.59	0.361	21.11	19.64-26.59	0.157	24.46	23.31-31.63	0.095	23.83	19.66-27.72	0.368
**Intensive pastures**	21.28	18.54-26.85	0.290	19.47	17.47-24.67	0.184	23.11	20.26-28.47	0.331	22.81	14.19-27.87	0.399	19.80	17.73-24.33	0.286
**Exotics species**															
**Native forest**	23.39	22.10-25.63	0.325	25.57	23.64-28.26	0.365	31.41	28.76-34.99	0.447	29.28	26.97-32.80	0.433	26.75	26.22-32.08	0.056
**Semi-natural pastures**	26.36	24.56-31.72	0.240	27.52	25.81-31.96	0.261	25.76	25.33-30.90	0.052	24.93	23.98-28.62	0.303	25.57	24.26-29.21	0.247
**Exotic forest**	37.95	37.23-45.04	0.070	36.10	36.45-43.88	>0.95	38.76	36.47-44.87	0.204	38.97	38.77-47.02	>0.95	43.52	42.16-53.99	0.095
**Intensive pastures**	25.24	24.18-29.07	0.197	24.27	23.89-27.86	0.102	27.86	26.74-32.73	0.192	27.44	26.86-31.80	0.089	23.92	24.19-28.01	>0.95

Table G. Degree of nestedness calculated for the four considered habitats (native forest, semi-natural pasture, exotic forest and intensive pasture) using presence–absence matrices of the raw data ($NODF_{raw}$), for the whole assemblages, and for indigenous and exotic species, separately. The 95% confidence interval of two null models after 1,000 permutations is indicated (PP is the proportional–proportional null model and RC is the null model controlling the passive sampling effect). All the significant NODF values indicate a lower degree of nestedness than those obtained in all the 1,000 null model permutations (P > 0.999; P > 0.95), indicating anti-nestedness.

	$NODF_{raw}$	Null models	95% IC	P
**Whole assemblages**				
**Native forest**	29.55	PP	28.46–32.34	0.301
		RC	55.33–63.01	>0.999
**Semi-natural pasture**	33.66	PP	31.94–38.00	0.250
		RC	57.70–64.65	>0.999
**Exotic forest**	32.86	PP	32.71–37.32	>0.95
		RC	51.79–61.20	>0.999
**Intensive pasture**	34.22	PP	34.07–37.95	>0.95
		RC	64.18–69.75	>0.999
**Indigenous species**				
**Native forest**	26.93	PP	26.58–29.89	0.080
		RC	54.84–62.55	>0.999
**Semi-natural pasture**	30.95	PP	28.66–34.51	0.404
		RC	57.94–68.31	>0.999
**Exotic forest**	26.75	PP	26.33–31.36	0.065
		RC	54.84–62.55	>0.999
**Intensive pasture**	28.75	PP	27.49–32.42	0.223

		RC	58.77–70.83	>0.999
**Exotics species**				
**Native forest**	33.94	PP	31.31–38.35	0.413
		RC	55.31–65.90	>0.999
**Semi-natural pastures**	37.41	PP	35.00–42.45	0.356
		RC	57.34–67.18	>0.999
**Exotic forest**	40.79	PP	39.65–46.52	0.108
		RC	54.11–65.90	>0.999
**Intensive pastures**	37.16	PP	36.92–41.01	0.051
		RC	66.31–72.06	>0.999

Table H.- Averaged pairwise values of β-diversity (β) based on a modification of the species replacement index proposed by [1] (see [2]), which was calculated per each considered habitat category for the whole epigean arthropod assemblages (All) and for the indigenous species alone. Wilcoxon Z values and P-values are also indicated for pairwise comparisons (N=120) between the whole assemblages and the indigenous species alone in the 10 rarefied matrices (ranged 1-10).

	Native forest	Semi-natural pasture	Exotic forest	Intensive pasture		Native forest	Semi-natural pasture	Exotic forest	Intensive pasture
1					6				
**β All**	0.69	0.64	0.59	0.69		0.66	0.66	0.62	0.65
**β Indigenous**	0.74	0.6	0.58	0.61		0.65	0.61	0.47	0.64
**Wilcoxon Z values**	4.18	2.01	0.33	3.06		0.95	2.27	4.94	0.38
**P**	<0.0001	<0.05	0.74	<0.01		0.341	<0.05	<0.00001	0.704
2					7				
**β All**	0.66	0.67	0.55	0.63		0.66	0.65	0.58	0.69
**β Indigenous**	0.67	0.64	0.5	0.49		0.65	0.54	0.58	0.62
**Wilcoxon Z values**	1.38	1.65	1.87	4.81		0.78	4.85	0.29	3.13
**P**	0.169	0.098	0.062	<0.00001		0.435	<0.00001	0.773	<0.01
3					8				
**β All**	0.64	0.65	0.55	0.68		0.62	0.65	0.62	0.64
**β Indigenous**	0.66	0.6	0.5	0.58		0.65	0.64	0.56	0.57
**Wilcoxon Z values**	1.31	2.32	1.83	4.03		2.5	0.27	1.83	3.6
**P**	0.192	<0.05	0.067	<0.0001		<0.05	0.789	0.068	<0.001

	4					9			
**β All**	0.61	0.65	0.55	0.62		0.65	0.61	0.58	0.63
**β Indigenous**	0.57	0.6	0.53	0.63		0.7	0.48	0.58	0.55
**Wilcoxon Z values**	1.99	2.4	0.36	0.72		4.23	5.33	0.17	3.12
**P**	<0.05	<0.05	0.719	0.471		<0.0001	<0.00001	0.864	<0.01
	5					10			
**β All**	0.62	0.59	0.57	0.62		0.64	0.66	0.54	0.64
**β Indigenous**	0.64	0.52	0.52	0.5		0.68	0.56	0.53	0.55
**Wilcoxon Z values**	1.3	3.11	1.5	3.73		3.12	4.5	0.73	3.37
**P**	0.195	<0.01	0.134	0.001		<0.01	<0.00001	0.469	<0.001

References

1. Williams PH (1996) Mapping variations in the strength and breadth of biogeographic transition zones using species turnover. Proc R Soc B: Biol Sci 263: 579-588.

2. Cardoso P, Borges PAV, Veech JA (2009) Testing the performance of beta diversity measures based on incidence data: the robustness to undersampling. Diversity Distrib 15: 1081-1090.
